# Potentiation of apoptosis in drug-resistant mantle cell lymphoma cells by MCL-1 inhibitor involves downregulation of inhibitor of apoptosis proteins

**DOI:** 10.1038/s41419-023-06233-w

**Published:** 2023-11-02

**Authors:** Yijing Li, Heng-Huan Lee, Vivian Changying Jiang, Yuxuan Che, Joseph McIntosh, Alexa Jordan, Jovanny Vargas, Tianci Zhang, Fangfang Yan, Margaret Elizabeth Simmons, Wei Wang, Lei Nie, Yixin Yao, Preetesh Jain, Michael Wang, Yang Liu

**Affiliations:** 1 Department of Lymphoma and Myeloma, The University of Texas MD Anderson Cancer Center, Houston, TX 77030 USA; 2 Department of Stem Cell Transplantation and Cellular Therapy, The University of Texas MD Anderson Cancer Center, Houston, TX 77030 USA

**Keywords:** Cancer therapy, Cancer models

## Abstract

Bruton’s tyrosine kinase inhibitors (BTKi) and CAR T-cell therapy have demonstrated tremendous clinical benefits in mantle cell lymphoma (MCL) patients, but intrinsic or acquired resistance inevitably develops. In this study, we assessed the efficacy of the highly potent and selective MCL-1 inhibitor AZD5991 in various therapy-resistant MCL cell models. AZD5991 markedly induced apoptosis in these cells. In addition to liberating BAK from the antiapoptotic MCL-1/BAK complex for the subsequent apoptosis cascade, AZD5991 downregulated inhibitor of apoptosis proteins (IAPs) through a BAK-dependent mechanism to amplify the apoptotic signal. The combination of AZD5991 with venetoclax enhanced apoptosis and reduced mitochondrial oxygen consumption capacity in MCL cell lines irrespective of their BTKi or venetoclax sensitivity. This combination also dramatically inhibited tumor growth and prolonged mouse survival in two aggressive MCL patient-derived xenograft models. Mechanistically, the augmented cell lethality was accompanied by the synergistic suppression of IAPs. Supporting this notion, the IAP antagonist BV6 induced dramatic apoptosis in resistant MCL cells and sensitized the resistant MCL cells to venetoclax. Our study uncovered another unique route for MCL-1 inhibitor to trigger apoptosis, implying that the pro-apoptotic combination of IAP antagonists and apoptosis inducers could be further exploited for MCL patients with multiple therapeutic resistance.

## Introduction

Mantle cell lymphoma (MCL) is a subtype of B-cell lymphoma that is incurable because patients frequently relapse from their therapies [1]. Bruton’s tyrosine kinase inhibitors (BTKi), such as ibrutinib, and CAR T-cell therapy have demonstrated clinical benefits in relapsed/refractory (R/R) MCL patients. However, intrinsic or acquired resistance occurs frequently [2, 3]. The disease prognosis for these R/R patients is very poor, justifying an urgent unmet clinical demand for novel therapeutic modalities [4].

The evasion of apoptosis that is associated with MCL pathogenesis and therapy resistance frequently involves dysregulated members of the B-cell lymphoma 2 (BCL-2) family [5]. The pro-survival members BCL-2 and myeloid leukemia 1 (MCL-1) have long been attractive targets because they are upregulated in diverse cancer types and confer drug resistance [6, 7]. The BCL-2 inhibitor venetoclax is efficacious in both BTKi-naive MCL patients (ORR = 75%) and BTKi-R/R MCL patients (ORR = 53%) [8, 9]. However, most MCL patients either have intrinsic resistance or develop acquired resistance to venetoclax eventually. A switch in apoptosis dependency from BCL-2 to other anti-apoptotic BCL-2 family members, such as MCL-1, contributes to venetoclax resistance in lymphoid malignancies [10, 11]. Additionally, MCL-1 transgenic mice often develop aggressive subtypes of B-cell lymphoma [12], and MCL-1 is critical for the sustained growth and survival of c-MYC driven lymphoma [13]. Collectively, MCL-1 is a therapeutic vulnerability for MCL.

Inhibitor of apoptosis proteins (IAPs) are a family of negative apoptosis regulators that interfere with the activated caspases to block apoptosis and contribute to pro-survival signaling pathways [14]. Eight IAPs have been identified in humans, with XIAP, c-IAP1, and c-IAP2 as prominent members [15]. Overexpression of IAP proteins in cancer has been associated with tumor progression and poor prognosis;[16, 17] therefore, targeting IAP proteins represents a promising therapeutic intervention in human malignancies.

Herein, we investigated the anti-MCL efficacy of the highly potent and selective MCL-1 inhibitor AZD5991 in various resistant MCL models. We found that AZD5991 induced dramatic MCL cell death, independent of resistance to ibrutinib and venetoclax. Moreover, the combination of AZD5991 with venetoclax was effective against therapy-resistant MCL models in vitro and in vivo. This enhanced cell lethality was accompanied by remarkable downregulation of IAPs, suggesting that development of AZD5991 plus venetoclax, or venetoclax plus IAP antagonist combinatorial treatments for therapy-resistant patients with MCL could improve treatment durability for this incurable disease.

## Materials and methods

### Reagents and cell lines

AZD5991 was obtained from AstraZeneca (Cambridge, UK). Venetoclax was purchased from Selleck Chemicals (Houston, TX, USA). S63845, MIK665 and BV6 were purchased from TargetMol Chemicals (Boston, MA, USA). The MCL cell lines Mino, Rec-1, JeKo-1, Z-138, and MAVER-1 were obtained from the American Type Culture Collection (Manassas, VA, USA). The venetoclax-resistant cell line Mino-VEN-R (Mino-venetoclax-R) was generated by chronic venetoclax treatment of parental Mino cells. JeKo-IBN-R (JeKo-ibrutinib-R) was generated from JeKo-1 cells treated with escalating doses of ibrutinib. The JeKo BTK KD_1 and JeKo BTK KD_2 cell lines were generated through BTK knockdown using CRISPR/Cas9 editing by the MD Anderson Core Facility and has been previously verified and published [18]. Cells tested negative for Mycoplasma contamination and were authenticated by short tandem repeat profiles. Cells were cultured in RPMI-1640 (Sigma-Aldrich, St Louis, MO, USA) supplemented with 10% heat-inactivated fetal bovine serum and penicillin-streptomycin solution (Mediatech, Inc., Manassas, VA, USA). All cell lines were maintained in a tissue culture incubator at 37 °C supplemented with 5% CO_2_. Antibodies to MCL-1, BCL-2, BCL-X_L_, GAPDH, poly (ADP-ribose) polymerase (PARP), BAK, BAX, NOXA, XIAP, c-IAP1, c-IAP2, c-MYC, and cleaved Caspase 3 (c-Caspase 3) were purchased from Cell Signaling Technology (Beverly, MA, USA). Antibodies to β-actin, MCL-1 and BCL-2 were purchased from Santa Cruz Technology (Dallas, TX, USA). Normal Rabbit Immunoglobulin (IgG) was purchased from PeproTech (Cranbury, NJ, USA).

### Reanalysis of previously published single-cell RNA sequencing data

To investigate MCL-1 expression at the single-cell level, we integrated two of our previously published single-cell RNA-seq datasets [19, 20]. We focused our analysis on three patient groups: ibrutinib-sensitive (IBN-S), ibrutinib-resistant (IBN-R), and ibrutinib-/CAR T dual-resistant (IBN-/CAR T-R). The raw count matrices from both datasets were integrated and preprocessed, including normalization, dimension reduction, and cell type annotation. Subsequently, we extracted the expression levels of the *MCL1* gene and assessed the significance of differences between two groups using the default Wilcoxon Rank Sum test.

### Cell viability assay

MCL cell lines were plated at 1 × 10^4^ per well in 96-well plates and treated with drug for 72 h. Drug concentrations ranged from 0.02 to 1000 nM. Cell viability was assessed using CellTiter-Glo (Promega, Madison, WI, USA). Primary MCL samples were obtained after informed consent, following Institutional Review Board-approved protocols and in accordance with the Declaration of Helsinki, from patients of the Department of Lymphoma and Myeloma at MD Anderson Cancer Center. Purified peripheral blood mononuclear cells were isolated from apheresis samples and treated with drug for 24 h. Dose–response curves for viability were plotted and analyzed for IC_50_ using GraphPad Prism. Data from the cell viability assays were normalized to samples with DMSO treatment.

### Apoptosis assay

MCL cells were seeded in 24-well plates overnight prior to treatment. The seeded cells were incubated with single agents or combinations for 24 h at 37 °C. After centrifugation for 5 min at 1500 rpm, cells were washed with PBS, then resuspended in annexin V binding buffer (BD Biosciences, Franklin Lakes, NJ, USA) and stained with annexin V-FITC (BD Biosciences) and propidium iodide (Invitrogen) for 15 min. Flow cytometric analyses were conducted with NovoCyte Flow Cytometer (ACEA Biosciences, San Diego, CA, USA).

### Western blotting assay

MCL cell lines were treated for 16 h with either single agent or their combination prior to harvest. Cells were then lysed on ice in lysis buffer (50 mmol/L HEPES pH 7.4, 150 mmol/L NaCl, 1% NP40, 1 mmol/L EDTA) containing a complete protease inhibitor cocktail (Roche) for 30 min, then centrifuged at 4 °C for 10 min at 14,000 g; 4 × loading buffer was added to the lysates after protein concentrations were measured. All original western blots are available in the Supplemental Material.

### siRNA transfection

MCL cell lines were electroporated following the protocol of kit V (Lonza, Cambridge, MA, USA) with the Amaxa nucleofector system according to the manufacturer’s instructions. MISSION® siRNA universal negative control #1 was used as control (Table S1). Cells were cultured in culture medium after electroporation at 37 °C for 48 h, then harvested and subjected to immunoblotting analysis.

### Co-immunoprecipitation (co-IP) assay

MCL cell lines were treated with AZD5991 or DMSO for 0.25, 2, 4, or 24 h. Cells were then harvested and lysed for 30 min in the aforementioned lysis buffer with the protease inhibitor cocktail. Protein concentration of the lysates was measured. 25 μg of protein lysate was loaded as input. To obtain IP samples, lysates were diluted to 1 mg/ml in 2 ml, then pre-cleared for 30 min using rotation at 4 ^o^C with a slurry of Protein A/G (1:1) magnetic beads (Thermo Fisher Scientific, Waltham, MA, USA). The pre-cleared lysates were incubated with anti-MCL-1 antibody (1:100, cat# 94296 S, Cell Signaling Technology) and protein A/G magnetic beads overnight at 4 °C with rotation. Beads were washed four times with cold lysis buffer, and the proteins were then eluted with 2× loading buffer. Input samples or IP samples were heated at 95 °C for 10 min after adding the loading buffer. Input samples for western blotting from MCL cell lines were probed with antibodies against MCL-1, BCL-2, BCL-X_L_, BAX, BAK, NOXA, c-Caspase 3, PARP, and β-actin. IP samples were probed with antibodies against MCL-1, BAK, BAX, and NOXA.

JeKo BTK KD_2 cells were used to detect the interaction between BAX with MCL-1 or BCL-2. Cells were processed as mentioned above. IP samples of BAX (1:50, cat# 2774 S, Cell Signaling Technology) or IgG (1:250, cat# 500-P00, PeproTech) were obtained as mentioned above after pre-clearing. 25 μg of protein lysate was loaded as input. Input and IP samples were probed with antibodies against MCL-1, BCL-2 and BAX.

### Oxygen consumption rate (OCR) assay

The OCR in intact cells was measured using an XF Cell Mito Stress Test kit (Agilent Technologies, Santa Clara, CA, USA). Cells were treated with AZD5991, venetoclax, or their combination at indicated concentrations for 2 h and then seeded in XF cell culture microplates. The OCR was measured by Seahorse XF96 flux analyzers. The basal level was attained by subtracting non-mitochondrial respiration and the maximal OCR was recorded after adding FCCP to induce maximal respiration. Detailed procedure was described previously [21].

### In vivo study

Mino-VEN-R mouse model was established by subcutaneously inoculating Mino-VEN-R cells into NSG mice. Patient-derived xenograft (PDX) mouse models were generated by inoculating freshly isolated PDX cells (from successfully established PDX) subcutaneously into NSG mice. When the tumors became palpable, the mice (*n* = 4 or 5 for each group) were treated with AZD5991 at 60 mg/kg (2-h split dose, iv) weekly, or S63845 at 30 mg/kg (iv) twice per week, or BV6 at 20 mg/kg (ip) (three times per week) or venetoclax orally at 5 mg/kg or 10 mg/kg once daily. Tumor volumes, survival duration, and body weight were monitored. Tumor diameters were measured biweekly starting on Day 0 of tumor cell inoculation until the maximal tumor diameter reached 15 mm, and tumor volumes were calculated as V = (L × W × W)/2. Mouse blood was collected on day 35 post-tumor cell inoculation. Human beta-2-microglobulin (β2M) level was detected by ELISA (Thermo Fisher Scientific) according to the manufacturer’s instructions.

### Statistical analyses

GraphPad Prism v9.0.0 (San Diego, CA, USA) was used to calculate statistical significance. Where appropriate, the data are presented as mean ± SD of triplicate samples. The IC_50_ values were calculated from at least three independent experiments and presented as mean ± SD. Comparison of differences between groups were conducted by Student’s *t*-test. Results were considered statistically significant at *p* < 0.05.

## Results

### AZD5991 restricts cell growth and induces apoptosis in MCL cells

**Fig. 1:**
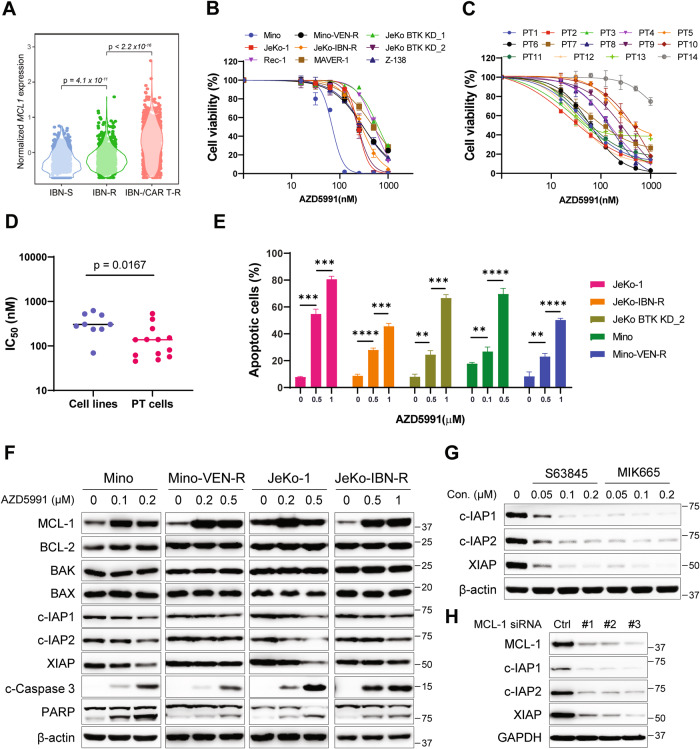
AZD5991 induced cytotoxicity and apoptosis in the ibrutinib- and/or venetoclax-resistant MCL cell lines and primary patient cells. **A** Violin plots show *MCL1* expression in ibrutinib-sensitive (IBN-S, *n* = 12), ibrutinib-resistant (IBN-R, *n* = 9) and ibrutinib-/CAR T dual-resistant (IBN-/CAR T-R, *n* = 5) MCL patient samples by single-cell RNA sequencing. Each dot represents a single cell. **B** Cell viability assay was conducted using the CellTiter-Glo cell viability assay (Promega) in the presence of dose gradients of AZD5991 in a panel of MCL cell lines. **C** Cell viability assay was performed in primary MCL cells after 24-hour treatment. **D** IC_50_ values of AZD5991 in MCL cell lines and primary cells. **E** Annexin V/PI cell apoptosis assay was performed in the indicated MCL cell lines. *p < 0.05, **p < 0.01, ***p < 0.001, ****p < 0.0001. **F** Western blot analyses after 16-hour treatment of AZD5991 at indicated concentrations; β-actin was used as the loading control. **G** IAPs expression in Mino cells treated with two additional MCL-1 inhibitors, S63845 and MIK665 for 16 h. **H** Expression of IAPs in Mino cells following electroporation with three MCL-1 siRNAs.

We first reanalyzed our previously published single-cell RNA sequencing datasets [19, 20], including a cohort of ibrutinib-sensitive, ibrutinib-resistant, and dual ibrutinib-/CAR T-resistant patient specimens, to interrogate the correlation between the *MCL1* expression and therapeutic resistance in MCL. The ibrutinib-resistant samples had higher *MCL1* expression compared to the ibrutinib-sensitive samples and the dual ibrutinib-/CAR T-resistant samples had the highest *MCL1* expression, implying a positive correlation between *MCL1* and therapeutic resistance (Fig. 1A). To determine whether vulnerability to AZD5991 correlates with the sensitivity to ibrutinib or venetoclax, we performed cell viability assays to test the in vitro efficacy of AZD5991 in a panel of MCL cell lines. MCL cell lines that were sensitive or resistant to ibrutinib or venetoclax demonstrated susceptibility to AZD5991 (*n* = 9, IC_50_ = 69.3 − 523.5 nM), and Mino exhibited a particularly high sensitivity to AZD5991 (Fig. 1B, D). The cytotoxicity of AZD5991 in a panel of MCL primary samples from patients who were naïve, sensitive, or resistant to single or multiple treatments of BTKi, venetoclax, and anti-CD19 CAR T-cell therapy was further evaluated (Table S2). Except for the aggressive cells from patient 14 (PT14) who relapsed after ibrutinib, venetoclax, and CAR T-cell therapy, AZD5991 effectively reduced cell viability in patient primary cells irrespective of the clinical resistance status, with IC_50_ values at the nanomolar range (IC_50_ = 45.14 – 533.5 nM) (Fig. 1C, D). Strikingly, AZD5991 demonstrated much higher potency in the primary cells than in the cell lines (Fig. 1D).

To investigate the mechanisms in overcoming resistance in MCL, we performed in vitro studies of JeKo-1 ibrutinib-sensitive cells, JeKo BTK KD_2 cells with intrinsic resistance to BTK inhibitors, and JeKo-IBN-R cells with acquired resistance to ibrutinib, Mino venetoclax-sensitive cells, and Mino-VEN-R cells with acquired resistance to venetoclax. AZD5991 effectively triggered cell apoptosis in these resistant cell lines in a dose-dependent manner, albeit with lower potency compared to the sensitive cell lines (Fig. 1E). Immunoblotting was conducted to investigate the impact of AZD5991 treatment on the apoptosis-related proteins, including BCL-2 family members and three predominant IAPs (XIAP, c-IAP1, and c-IAP2). As previously reported [22], AZD5991 increased MCL-1 levels, whereas BCL-2 remained largely unchanged. Mechanistically, AZD5991 greatly induced cleavage of caspase 3 and PARP, as well as downregulation of IAP proteins, to facilitate the activation of apoptotic cascades, irrespective of whether the cells are sensitive or resistant to ibrutinib or venetoclax (Fig. 1F).

To validate the influence of MCL-1 on the downregulation of IAPs, we examined the effects of two additional MCL-1 inhibitors, namely S63845 and MIK665. Consistent with AZD5991, S63845 and MIK665 resulted in a downregulation of IAPs in Mino and JeKo-1 cells (Fig. 1G, Fig. S1A). In addition, genetic perturbation through siRNA-mediated knockdown of MCL-1 robustly decreased the expression of IAPs in JeKo-1 and Mino cells, which provides further evidence for the critical role of MCL-1 in the regulation of IAPs (Fig. 1H, Fig. S1B).

### AZD5991 activates the BAK-dependent mitochondrial apoptotic pathway

**Fig. 2:**
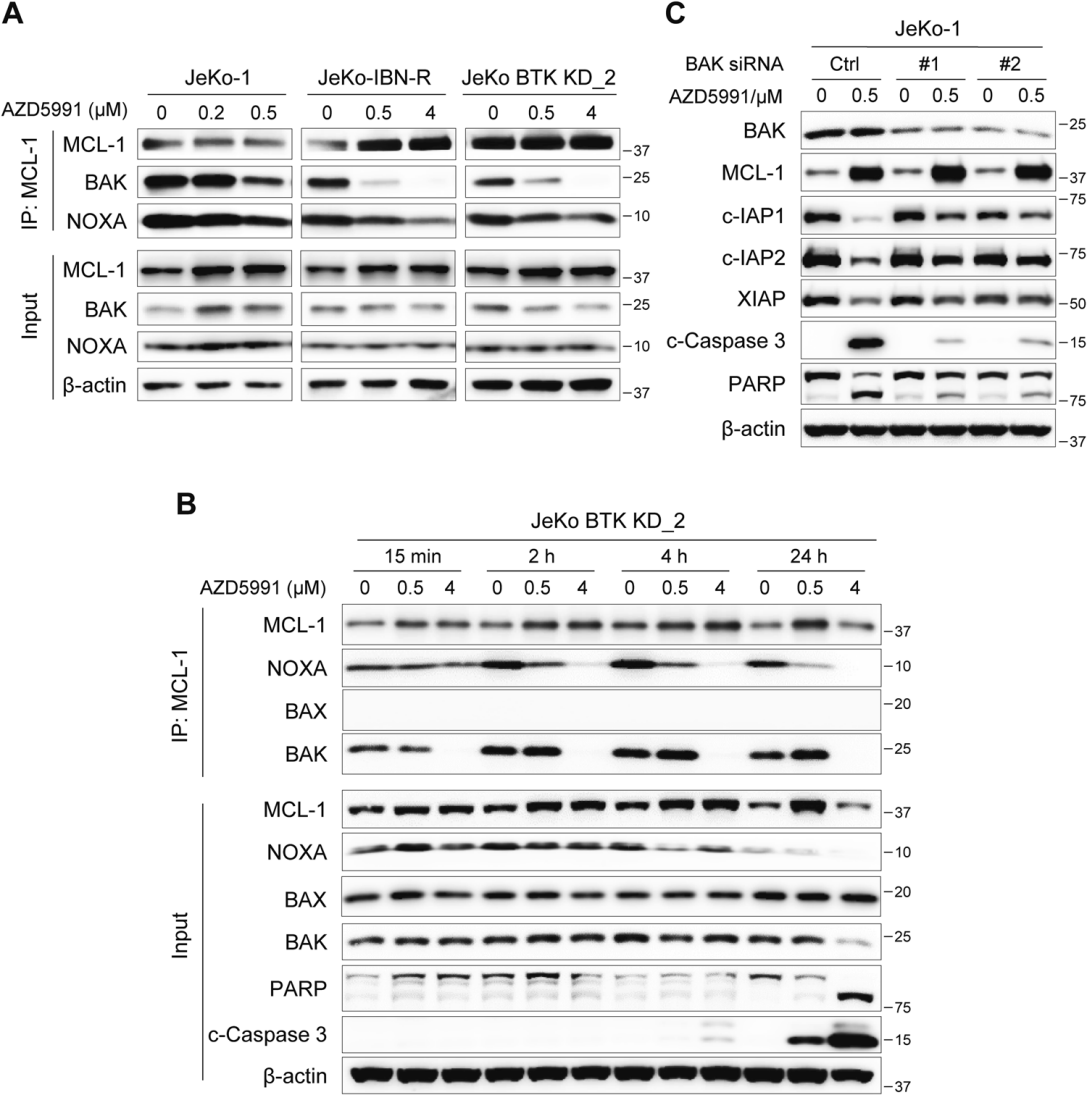
AZD5991 induced apoptosis by displacing BAK and NOXA from MCL-1/BAK complex or MCL-1/NOXA complex. **A** Whole cell extract (bottom) and co-IP of MCL-1 (top) was conducted using lysates from JeKo-1, JeKo-IBN-R, or JeKo BTK KD_2 cells after a 15-min treatment with AZD5991. **B** Time-dependent co-IP assay of MCL-1 with AZD5991 treatment in JeKo BTK KD_2 cells. **C** JeKo-1 cells were electroporated with BAK-targeted siRNAs followed by a subsequent 24-h exposure to AZD5991.

To interrogate mitochondrial dynamics during apoptosis in response to AZD5991, co-IP assays were performed to determine the interactions of MCL-1 with NOXA and BAK in JeKo-1 and its isogenic cell lines JeKo-IBN-R and JeKo BTK KD_2 cells. Treatment with AZD5991 for 15 min dissociated BAK and NOXA from MCL-1 in a dose-dependent manner (Fig. 2A), and the apoptotic hallmarks including caspase 3 activation and PARP cleavage were induced after 2 h of treatment (Fig. S2A, B). Time-dependent co-IP assays in JeKo BTK KD_2 cells further confirmed these findings, and the dissociation of BAK and NOXA from MCL-1 persisted after 24 h treatment (Fig. 2B). Despite the pretreatment with protein synthesis inhibitor cycloheximide, AZD5991 consistently reduced the BAK levels associated with MCL-1, implying that AZD5991 directly affected the interaction between BAK and MCL-1 without requiring de novo protein synthesis for the effect (Fig. S2C). Intriguingly, no interaction between MCL-1 and BAX was observed in these experiments, and this notion was further supported by the detected interaction of BAX with BCL-2 instead of MCL-1 in untreated JeKo BTK KD_2 cells (Fig. S2D). Based on these findings, it can be inferred that AZD5991 induced cell apoptosis by releasing BAK from the MCL-1/BAK complex, subsequently activating the BAK-mediated mitochondrial apoptotic pathway.

To investigate the dependence of IAPs downregulation on BAK, electroporation of siRNA was employed to knockdown BAK expression in JeKo-1 cells before AZD5991 treatment. As shown in Fig. 2C, knockdown of BAK effectively rescued the degradation of IAP proteins and reduced cell death, as evidenced by decreased levels of cleaved caspase 3 and PARP, indicating that downregulation of IAP proteins induced by AZD5991 is BAK-dependent.

### Combination of AZD5991 and venetoclax synergistically induces robust cytotoxicity

**Fig. 3:**
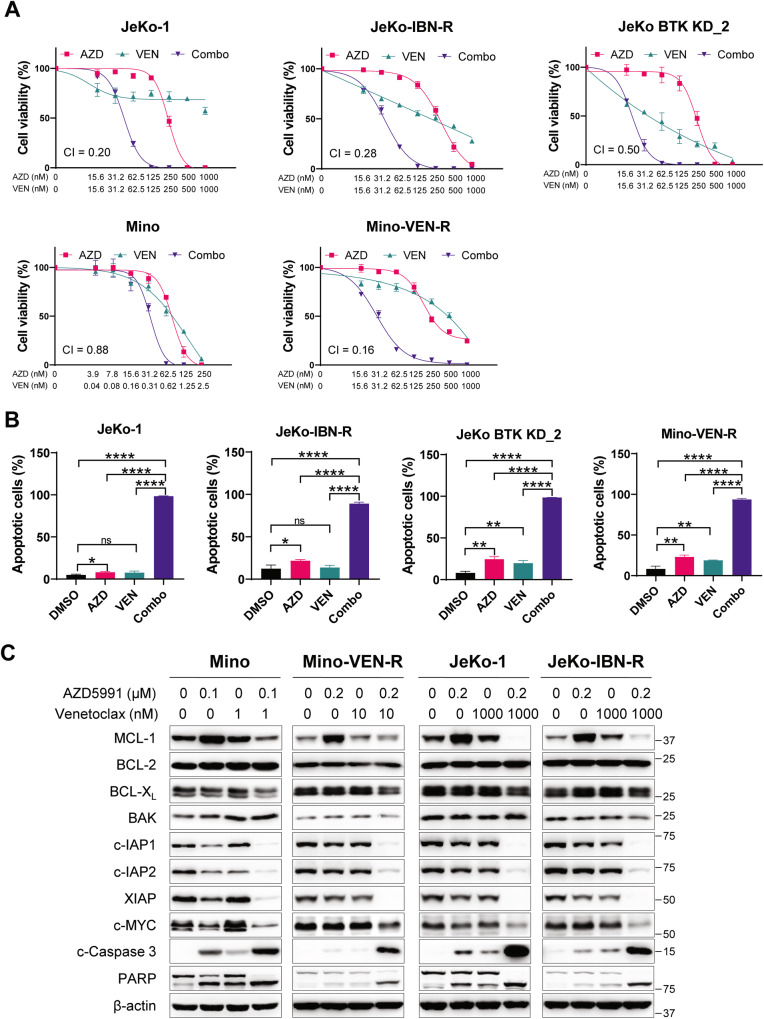
The AZD5991-venetoclax combination induced synthetic lethality and enhanced cell apoptosis in ibrutinib- and/or venetoclax-sensitive and -resistant cell lines. **A** Cell viability assay was examined after 72-h co-treatment with AZD5991 and venetoclax in MCL cell lines by the CellTiter-Glo cell viability assay (Promega). *p < 0.05, **p < 0.01, ***p < 0.001, ****p < 0.0001. **B** Annexin V/PI apoptosis assessments were performed following combinatorial AZD5991-venetoclax treatment in MCL cell lines. **C** Western blot analyses in JeKo-1, JeKo-IBN-R, Mino and Mino-VEN-R cells treated with AZD5991, venetoclax, or the combination for 16 h. AZD AZD5991; VEN venetoclax, Combo combination.

Given that MCL-1 overexpression confers resistance to venetoclax and that there is favorable synergy in combinatorial targeting of MCL-1 and BCL-2 in multiple myeloma and acute myeloid leukemia [23–26], we hypothesized that AZD5991 may restore venetoclax efficacy and induce synergistic lethality in venetoclax-resistant MCL cells. To assess the anti-MCL synergy of the AZD5991-venetoclax combination, we performed cell viability and apoptosis assays in the aforementioned MCL cell lines. The combined treatment yielded robust and synergistic cytotoxicity in all five cell lines independent of their sensitivity to venetoclax or ibrutinib, with synergy being indicated by a combination index (CI) < 1 (Fig. 3A). The synergistic effect was also shown by enhanced apoptosis accompanied by significantly activated caspase 3 and PARP cleavage (Fig. 3B, C), while BCL-2 and BCL-X_L_ remained largely unaffected. Strikingly, the anti-apoptotic MCL-1 was synergistically diminished in the combination treated cells, accompanied by significant downregulation of the negative regulator of apoptosis proteins, XIAP, c-IAP1, and c-IAP2, indicative of augmented apoptosis processing. Moreover, compared to DMSO control and both single treatments, the combination eradicated the expression of oncogenic c-MYC, which contributes to intrinsic ibrutinib resistance in MCL [27].

**Fig. 4:**
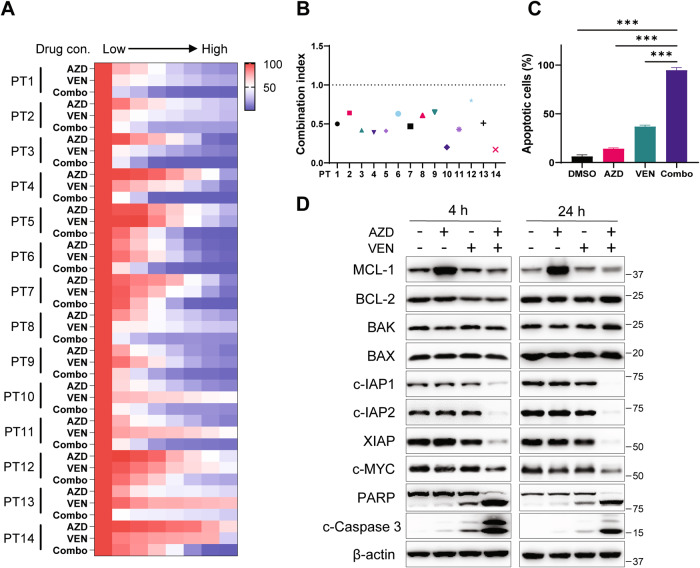
AZD5991 in combination with venetoclax triggered enhanced cell death in primary MCL patient cells. **A** The heatmap depicts cell viability after 24-h co-treatment with AZD5991 and venetoclax in primary cells from 14 MCL patients. **B** The graph summarizes the CI values for primary cells from the 14 patients. **C** Annexin V/PI apoptosis assay after 24-h exposure to AZD5991 (0.2 µM), venetoclax (0.2 µM), or the combination in PT14 cells, ***p < 0.001. **D** Expression of c-MYC and pro-apoptotic markers were detected by western blot after 4-h and 24-h treatment of AZD5991 (0.2 μM) and venetoclax (0.2 μM) against PT14 cells. AZD AZD5991; VEN venetoclax, Combo combination.

The augmented potency prompted us to further investigate the combination in a panel of MCL patient primary samples, including treatment-naïve, ibrutinib- or venetoclax-sensitive, and the BTKi- and/or venetoclax-resistant samples (Fig. 4A, Table S2). As expected, the combination synergistically reduced the primary cell viability with CI values ranging from 0.17 to 0.80 (*n* = 14, Fig. 4A, B). The exceptionally aggressive MCL tumor cells from PT14, which is resistant to multiple current clinical therapies, were resistant to single treatments with either venetoclax or AZD5991 but was sensitive to their combination, so it was chosen for further mechanistic study. Although both single treatments induced moderate apoptosis, the combination triggered dramatic cell death, with remarkable activation of caspase 3 and PARP cleavage (Fig. 4C, D). In agreement with the apoptotic phenotype in cell lines, the levels of pro-survival MCL-1, XIAP, c-IAP1, c-IAP2, and c-MYC were all decreased in response to 24 h treatment with the combination.

### Combinatorial AZD5991 and venetoclax has a synergistic effect in suppressing oxygen consumption rate (OCR) in both ibrutinib- or venetoclax-sensitive and -resistant MCL cells

**Fig. 5:**
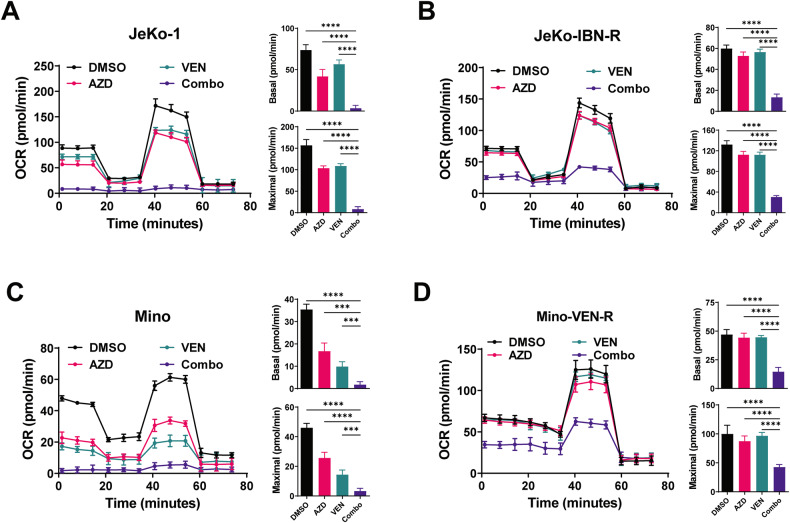
Seahorse analysis of mitochondrial oxygen consumption rate (OCR) in MCL cells. **A** OCR of JeKo-1 cells treated with AZD5991 (0.2 µM), venetoclax (1 µM), and their combination. **B** OCR of JeKo-IBN-R cells treated with AZD5991 (1 µM), venetoclax (2 µM), and their combination. **C** OCR of Mino cells treated with AZD5991 (0.1 µM), venetoclax (5 nM), and their combination. (D) OCR of Mino-VEN-R cells treated with AZD5991 (2 µM), venetoclax (0.2 µM), and their combination. AZD AZD5991; VEN venetoclax, Combo combination. *p < 0.05, **p < 0.01, ***p < 0.001, ****p < 0.0001.

Dysregulated mitochondrial oxidative phosphorylation (OXPHOS) contributes to both venetoclax resistance and ibrutinib resistance in lymphoma [21, 23]. Given the emerging evidence for MCL-1 in modulating mitochondrial dynamics and cell metabolism [28–31], we measured mitochondrial respiration upon the combination treatment using Seahorse metabolic flux assay in JeKo-1, JeKo-IBN-R, Mino, and Mino-VEN-R cell lines. As shown in Fig. 5, AZD5991 decreased both basal and uncoupled maximal respiration in sensitive JeKo-1 and Mino cells, while only maximal OCR was suppressed in the two resistant cell lines. Knockdown of BAK mediated by siRNA compromised the AZD5991-induced OCR reduction relative to that in control siRNA cells, supporting the notion that the alterations in OCR triggered by AZD5991 operated through a BAK-dependent mechanism (Fig. S3A, B). Remarkably, combination of AZD5991 and venetoclax dramatically reduced both basal and maximal OCR levels irrespective of resistance status, indicating a decreased capacity of mitochondrial respiration that is involved in the enhanced susceptibility to AZD5991.

### Combinatorial AZD5991 and venetoclax overcomes resistance to targeted and CAR T-cell therapy in MCL mouse models

**Fig. 6:**
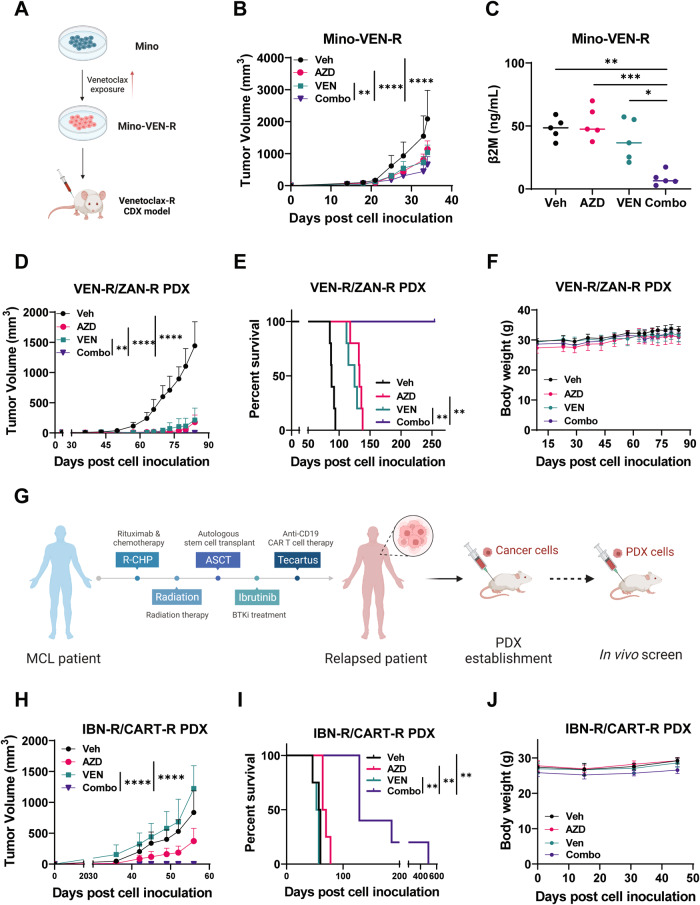
Combination of AZD5991 and venetoclax yielded anti-MCL synergy in therapy-resistant mouse models. **A** The schematic illustrates the generation of the venetoclax-resistant cell line xenograft (CDX) mouse model. **B** The mice were treated with vehicle (*n* = 5), venetoclax (*n* = 5, 10 mg/kg, oral, daily), AZD5991 (*n* = 5, 60 mg/kg, 2-h split dose, iv, weekly) or the combination therapy. Tumor volume was calculated as V = (L × W × W) / 2. **C** The graph shows the amount of β2M detected in the mouse serum on day 35 post-tumor cell inoculation. **D** A dually venetoclax- and zanubrutinib-resistant (VEN-R/ZAN- R) PDX mouse model was established to assess the synergism of combination AZD5991 and venetoclax. Freshly isolated PDX cells from the previous generation were inoculated subcutaneously into 8-week-old NSG mice. When tumors became palpable, the mice were treated with vehicle (*n* = 5), venetoclax (*n* = 5, 10 mg/kg, oral, daily), AZD5991 (*n* = 5, 60 mg/kg, 2-hour split dose, iv, weekly), or the combination. Tumor volume was calculated as above. **E** Survival curves of mice transplanted with VEN-R/ZAN-R PDX cells treated with the indicated drugs. **F** Mice body weights were plotted. **G** The graph illustrates the patient treatment history and the PDX establishment platform. **H** When tumors became palpable, the mice were treated with vehicle (*n* = 4), venetoclax (*n* = 4, 5 mg/kg, oral, daily), AZD5991 (*n* = 4, 60 mg/kg, 2-h split dose, iv, weekly), or the combination (*n* = 5). Tumor volume was calculated as V = (L × W × W) / 2. **I** Survival duration of mice post-tumor inoculation. **J** Mouse body weights during the treatment period. Veh Vehicle; AZD AZD5991; VEN venetoclax; Combo combination. *p < 0.05, **p < 0.01, ***p < 0.001, ****p < 0.0001.

To evaluate combinatorial AZD5991 and venetoclax in vivo, a venetoclax-resistant cell line-derived xenograft (CDX) mouse model was established by inoculation of Mino-VEN-R cells into immunodeficient NSG mice (Fig. 6A). Treatment of mice with AZD5991 alone reduced subcutaneous tumor volume; however, serum level of tumor burden marker β2M on day 35 showed that the tumor expansion in peripheral blood was not reduced. Impressively, the AZD5991-venetoclax combination significantly reduced both the subcutaneous tumor burden and decreased the β2M levels (Fig. 6B, C) as compared with both single treatment groups (*n* = 5, *p* = 0.0002 for AZD5991, *p* = 0.0191 for venetoclax), showing the superior in vivo anti-tumor activity of the combination. Moreover, mice tolerated the combinatorial treatment without body weight loss (data not shown). To determine the safety of simultaneously targeting MCL-1 and BCL-2, we assessed the MCL-1 inhibitor S63845, whose affinity for mouse MCL-1 is higher than that of AZD5991, in combination with venetoclax in the Mino-VEN-R mouse model. As anticipated, we observed synergistic effectiveness against MCL. No significant weight loss or hair loss were observed in the mice with this combination (Fig. S4).

The AZD5991 and venetoclax combination was further tested in a PDX mouse model established from a patient with dual resistance to venetoclax and the BTKi zanubrutinib (VEN-R/ZAN-R). AZD5991 and venetoclax, either alone or together, reduced tumor burden and prolonged mouse survival significantly compared to vehicle (Fig. 6D, E). Strikingly, mice treated with the AZD5991-venetoclax combination showed a significant delay in tumor growth, with 100% survival beyond 200 days after xenografting (Fig. 6E). Mice exhibited no weight loss during the entire treatment period (Fig. 6F). Collectively, these results indicate that the AZD5991-venetoclax combination holds promise in overcoming venetoclax and BTKi resistance in MCL.

We further investigated the combination in an aggressive PDX mouse model from an MCL patient with dual ibrutinib- and anti-CD19 CAR T-cell therapy resistance (IBN-R/CAR T-R) (Fig. 6G). Compared to vehicle, AZD5991 as single agent exhibited moderate tumor inhibitory activities, while venetoclax did not show any tumor inhibitory effects. Remarkably, the combination therapy completely suppressed the tumor growth up to 60 days post inoculation (Fig. 6H). In addition, the combination treatment prolonged mouse survival to a greater extent than AZD5991 single agent alone, with a median survival of 129 days versus 70 days for the AZD5991 group (Fig. 6I). All mice tolerated the drug treatments without developing symptoms indicative of physical stress, such as weight loss (Fig. 6J). The results clearly suggest that combinatorial AZD5991 and venetoclax is a potentially effective regimen for treatment of CAR-T resistance in MCL.

### Targeting IAP proteins can be exploited to enhance the pro-apoptotic stimuli

**Fig. 7:**
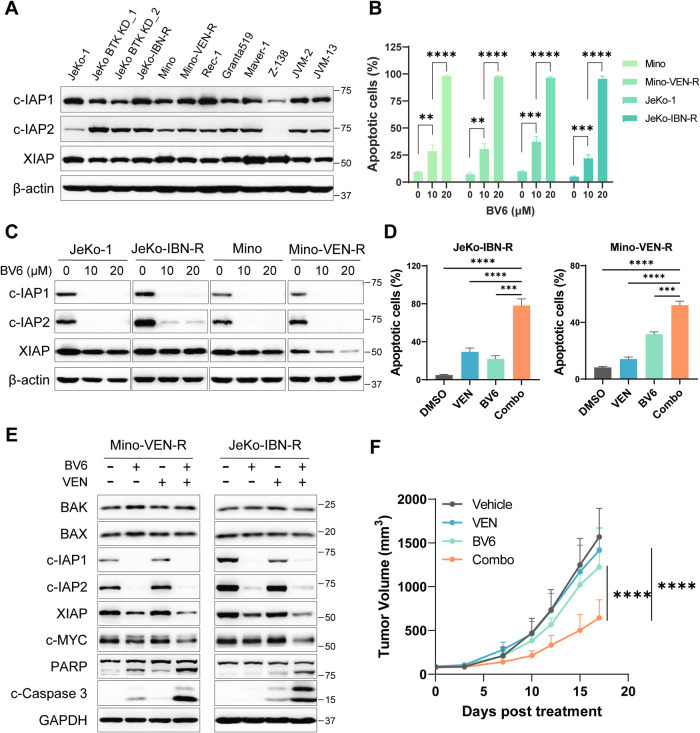
The IAP antagonist BV6 sensitized resistant MCL cells to venetoclax-induced apoptosis. **A** Western blot analyses of IAP family proteins across MCL cell lines. **B** Annexin V/PI apoptosis assay was conducted after 24-h treatment of BV6 against MCL cells. **C** MCL cell lines were treated with BV6 at indicated concentrations for 4 h, followed by detection of the IAP expression. **D** MCL cells were treated with BV6 (10 µM) in combination with venetoclax (0.1 µM for Mino-VEN-R and 2 µM for JeKo-IBN-R) for 24 h, cell apoptosis population was determined by flow cytometry. **E** Expression of IAPs and apoptosis-related proteins were measured after 16-hour treatment of the BV6 (10 µM) and venetoclax (0.1 µM for Mino-VEN-R and 2 µM for JeKo-IBN-R) combination by immunoblotting. **F** The mice were treated with vehicle (*n* = 4), venetoclax (*n* = 4, 5 mg/kg, oral, daily), BV6 (*n* = 4, 20 mg/kg, ip, three times per week), or the combination therapy (*n* = 4). Tumor volume was calculated as V = (L × W × W) / 2. Veh Vehicle; VEN venetoclax, Combo combination. *p < 0.05, **p < 0.01, ***p < 0.001, ****p < 0.0001.

In addition to the canonical pro-apoptotic role for AZD5991, the amplified apoptotic signal via IAP downregulation prompted us to examine IAP antagonists in MCL. The protein expression of the three prominent IAPs, XIAP, c-IAP1, and c-IAP2, was measured by western blotting in a panel of MCL cell lines. XIAP was widely expressed with altered expression patterns across the cell lines, whereas considerable variation was observed for c-IAP1 and c-IAP2 expression levels (Fig. 7A).

Extensive studies are being carried out to develop IAP antagonists that mimic endogenous IAP antagonist SMAC (second mitochondria-derived activator of caspase), which blocks caspase activation by disrupting interaction to XIAP, c-IAP1, and c-IAP2 [32]. The SMAC-mimetic IAP antagonist BV6 [33, 34] was selected in this study to evaluate the anti-MCL efficacy with our resistant cell models. BV6 induced strong apoptosis in a dose-dependent manner against both drug-resistant MCL cell lines and the aggressive PT14 cells (Figs. 7B, S5A). Four-hour treatment with BV6 markedly diminished the expression of c-IAP1 and c-IAP2 (Fig. 7C). To investigate whether BV6 could sensitize resistant MCL cells to pro-apoptotic stimuli, the two resistant cell lines as well as the aggressive tumor cells from PT14 were treated with BV6 in combination with venetoclax. As expected, BV6 significantly enhanced the pro-apoptotic effects of venetoclax (Figs. 7D, S5B). Comparable to AZD5991 plus venetoclax, BV6 plus venetoclax demonstrated a synergistic effect to reduce the expression of IAPs (especially XIAP) and c-MYC, and enhanced the activation of caspase 3 and PARP in the two resistant MCL cell lines (Fig. 7E). Subsequent evaluation in the Mino-VEN-R xenograft model revealed that the BV6 plus venetoclax combination led to a significant reduction in tumor volume, while either single agent had limited efficacy (Fig. 7F).

## Discussion

As an antiapoptotic protein, MCL-1 has become an attractive target and is implicated with venetoclax resistance in hematologic malignancies [7], motivating us to investigate AZD5991 alone and in combination with venetoclax in our resistant MCL models. Our in vitro screening revealed that MCL cell lines with either intrinsic or acquired resistance to ibrutinib or venetoclax demonstrated susceptibility to AZD5991 within the nanomolar range; its efficacy was confirmed in a panel of primary MCL cells irrespective of the status of their clinical resistance to ibrutinib, venetoclax, or even CAR T-cell therapy. Further, significant anti-MCL synergy was observed for the AZD5991-venetoclax combination in these resistant cell models. The combined treatment significantly reduced the tumor burden and prolonged survival in three aggressive mouse xenograft models (Mino-VEN-R CDX, VEN-R/ZAN-R PDX and IBN-R/CAR T-R PDX). Together, these findings pave the way for future investigation of combining BCL-2 and MCL-1 blockade to overcome multiple resistant MCL phenotypes, including those relapsed from anti-CD19 CAR T-cell therapy.

Metabolic reprogramming toward OXPHOS was implicated in both ibrutinib resistance in MCL [21] and venetoclax resistance in lymphoid malignancies [21, 23], confirming OXPHOS as an attractive target for MCL. MCL-1 was reported to positively regulate the OXPHOS and promote chemoresistance in cancer [31]. Especially but not solely in combination with venetoclax, dramatically reduced OCR at least partially accounts for the capability of AZD5991 and venetoclax combination to overcome therapeutic resistance in MCL.

MCL has a limited number of established cell lines to cover the full spectrum of phenotypes found in the clinic. Accordingly, we included several cell lines derived from those with differing therapy resistances, along with many patient-derived primary samples with multi-resistance phenotypes. Inclusion of these additional MCL cell lines and patient samples will certainly facilitate the translation from preclinical study to clinical outcome.

Evading apoptosis is a cancer hallmark. To date, several IAP antagonists have entered clinical trials, and are being investigated in combinatorial protocols [35]. The BAK-dependent reduction of IAPs by AZD5991 and two other MCL-1 inhibitors highlighted an intriguing regulation of IAPs by MCL-1 (Fig. S6). This finding warrants further investigation to elucidate underlying mechanisms in future studies. Moreover, it indicates that IAP antagonists or degraders could be exploited in combinatorial therapies to sensitize resistant MCL cells to pro-apoptotic therapeutics. Regarding the aggressive PT14 cells (Fig. 4D), we observed a reduction of IAPs only when treated with the combination, which could be attributed to the highly aggressive nature of this patient tumor. Nonetheless, it supports the notion that the therapeutic synergy is achieved when IAPs are efficiently downregulated, highlighting the potential significance of IAPs in the therapeutic efficacy of BCL-2 and MCL-1 blockade. Mechanistically, IAP antagonists can lower the cell death-inducing threshold when combining with apoptosis inducers like venetoclax, providing another reason to explore the pro-apoptotic combination by inhibiting both IAP and BCL-2 to overcome multiple resistance to targeted and CAR T-cell therapy in MCL.

### Supplementary information

Supplementary information

Original Data File

Reproducibility checklist.

## Data Availability

The original data and protocols can be obtained upon reasonable request.
