# Supplementary material for: Potentiation of apoptosis in drug-resistant mantle cell lymphoma cells by MCL-1 inhibitor involves downregulation of inhibitor of apoptosis proteins
Source: Cell Death Dis. 2023 Nov 2;14(11):714. doi: 10.1038/s41419-023-06233-w (PMC10622549; doi:10.1038/s41419-023-06233-w)
Supplement: Supplementary file 1 — Supplementary information [file 41419_2023_6233_MOESM1_ESM.docx]

**Potentiation of apoptosis in drug-resistant mantle cell lymphoma cells by MCL-1 inhibitor involves downregulation of inhibitor of apoptosis proteins**

Yijing Li^1^, Heng-Huan Lee^1^, Vivian Changying Jiang^1^, Yuxuan Che^1^, Joseph McIntosh^1^, Alexa Jordan^1^, Jovanny Vargas^1^, Tianci Zhang^1^, Fangfang Yan^1^, Margaret Elizabeth Simmons^1^, Wei Wang^1^, Lei Nie^1^, Yixin Yao^1^, Preetesh Jain^1^, Michael Wang^1,2,^, and Yang Liu^1,*^

**Supplementary Tables**

**Table S1**. MCL-1 siRNA sequences used for transient MCL-1 knockdown.

|  | **Company** | **Product Number** |
| --- | --- | --- |
| siRNA universal negative control #1 | Sigma-Aldrich | SIC001 |
| MCL-1 siRNA#1 | Sigma-Aldrich | SASI_Hs01_00162656 |
| MCL-1 siRNA#2 | Sigma-Aldrich | SASI_Hs01_00162657 |
| MCL-1 siRNA#3 | Sigma-Aldrich | SASI_Hs01_00162658 |
| BAK siRNA#1 | Sigma-Aldrich | SASI_Hs02_00331326 |
| BAK siRNA#2 | Sigma-Aldrich | SASI_Hs01_00331327 |

**Table S2.** Patient characteristics for the samples involved in the study.

| **Sample ID** | **Gender** | **Age** | **Treatment history** | **Mutations** | **Cytogenetics** |
| --- | --- | --- | --- | --- | --- |
| PT1 | M | 80 | Naïve | *TP53*, *MYC*, *ATM* | IGH/CCND1/MYEOV Rearrangement |
| PT2 | M | 65 | Naïve | *TP53*, *MYC* | Complex karyotype with t(11;14). IGH/CCND1/MYEOV Rearrangement |
| PT3 | M | 71 | Naïve | *TP53* | Complex karyotype with t(11;14), positive t(11;14)/IGH-CCND1 |
| PT4 | M | 89 | Naïve | ND | Positive for CCND1-IGH Dual Fusion Signals; t(11;14). IGH/CCND1/MYEOV Rearrangement |
| PT5 | M | 40 | Venetoclax-S | ND | Abnormal t(11;14) and DEL(13Q). IGH/CCND1/MYEOV Rearrangement |
| PT6 | M | 50 | Ibrutinib-S | *TP53*, MYC | IGH/CCND1/MYEOV Rearrangement |
| PT7 | M | 53 | Ibrutinib-S  Venetoclax-S | ND | Normal male karyotype |
| PT8 | F | 63 | Venetoclax-S | *TP53*, *MYC* | Complex karyotype with t(11;14) |
| PT9 | M | 43 | Venetoclax-S | *MYC* | Normal male karyotype |
| PT10 | M | 62 | Ibrutinib-R | *ATM* | IGH/CCND1/MYEOV Rearrangement |
| PT11 | M | 74 | Acalabrutinib-R Venetoclax-R | *MYC* | IGH/CCND1/MYEOV Rearrangement |
| PT12 | F | 58 | Ibrutinib-R  Anti-CD19 CAR T-R | *TP53*, *MYC* | IGH/CCND1/BCL-1 Rearrangement |
| PT13 | M | 74 | Ibrutinib-R  Zanubrutinib-R  Venetoclax-R | *TP53*, *ATM* | IGH/CCND1/MYEOV Rearrangement |
| PT14 | M | 74 | Acalabrutinib-R Venetoclax-R  Anti-CD19 CAR T-R | *MYC*, *ATM* | IGH/CCND1/MYEOV Rearrangement |

S, Sensitive; R, Resistant; ND, Not detected.

**Supplementary Figures**


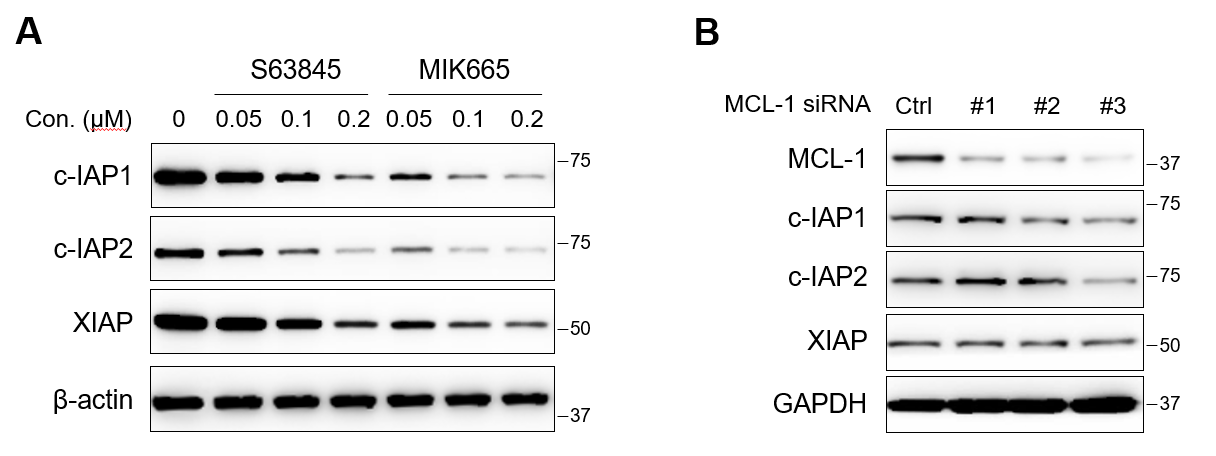


**Figure S1.** Expression levels of IAPs in JeKo-1 cells following treatment with two additional MCL-1 inhibitors (A) or siRNA by electroporation (B).


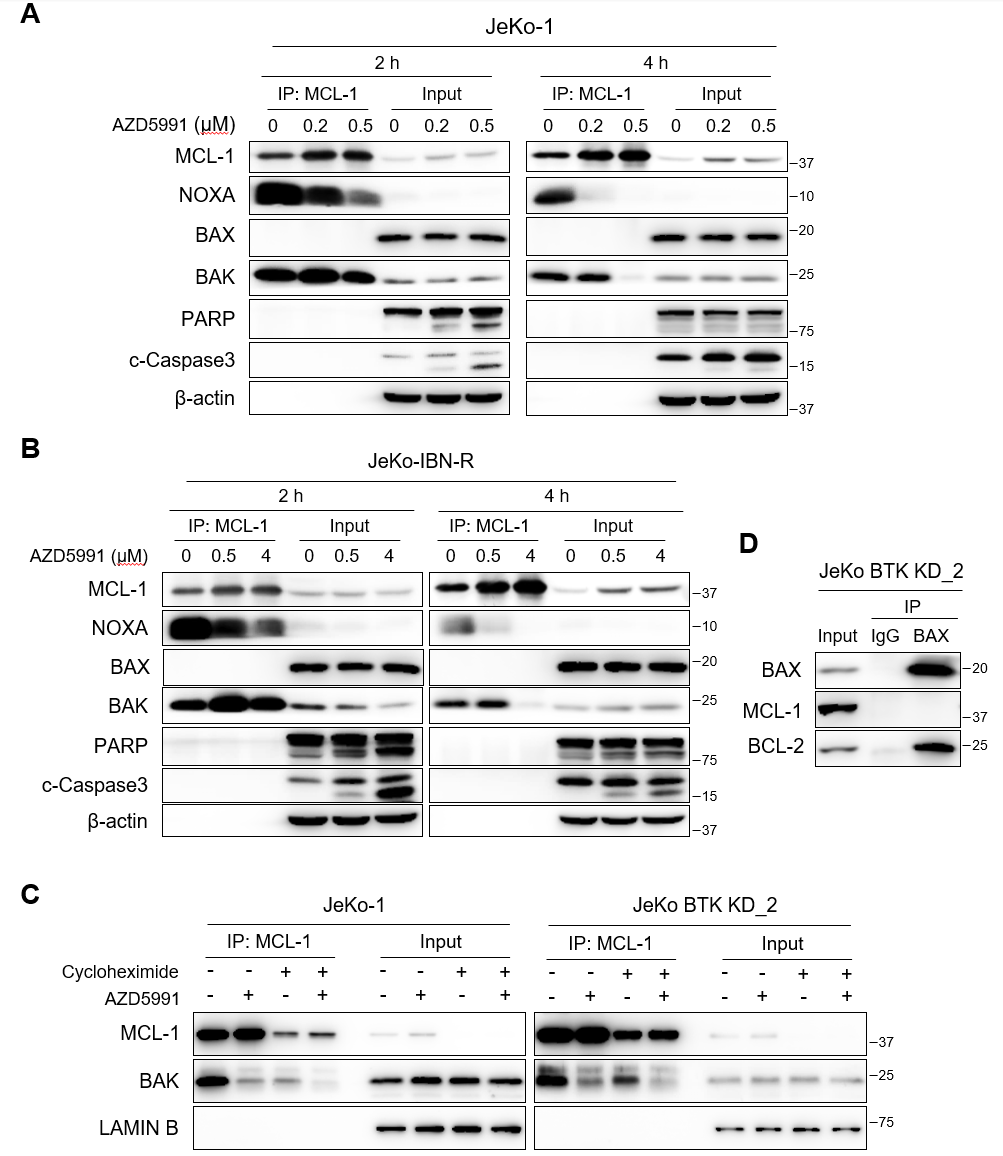


**Figure S2.** AZD5991 induced apoptosis by displacing BAK and NOXA from the MCL-1/BAK and MCL-1/NOXA complex. (A) Co-IP analysis of MCL-1 in JeKo-1 cells after 2- or 4-h treatment with AZD5991 at indicated concentrations. (B) Co-IP analysis of MCL-1 in JeKo-IBN-R cells after 2- or 4-h treatment with AZD5991 at indicated concentrations. (C) JeKo-1 and JeKo BTK KD_2 cells were pretreated with cycloheximide (25 µg/ml) for 1 h, followed by a 20-minute treatment with AZD5991. (D) Co-IP analysis of BAX in JeKo BTK KD_2 cells.


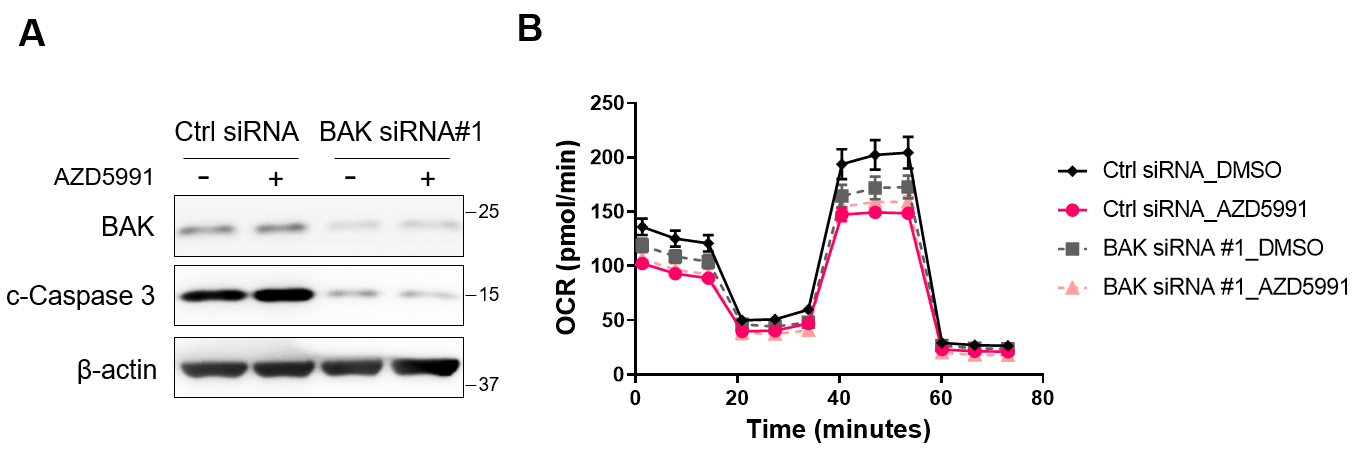


**Figure S3.** Seahorse analysis of mitochondrial oxygen consumption rate (OCR) in Mino cells. (A) siRNA-mediated BAK knockdown in Mino cells. (B) Mino cells were electroporated with BAK-targeted or control siRNAs followed by 2 h exposure to AZD5991 (0.1 µM).


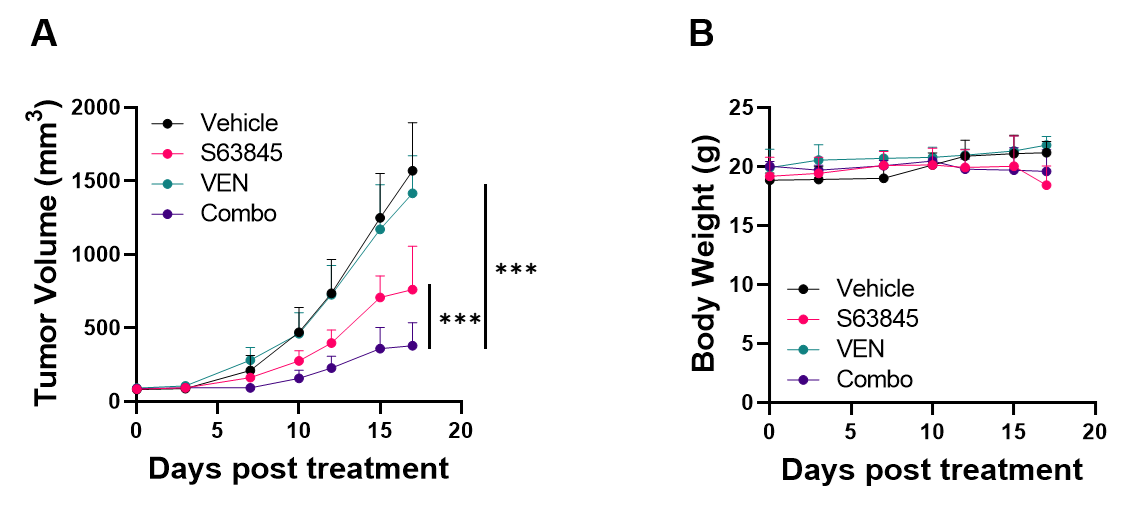


**Figure S4.** Combination of S63845 and venetoclax yields anti-MCL synergy in Mino-VEN-R xenograft mouse models. (A) When tumors became palpable, the mice were treated with vehicle (n = 4), venetoclax (n = 4, 5 mg/kg, oral, daily), S63845 (n = 4, 30 mg/kg, iv, twice per week) or the combination therapy (n = 4). Tumor volume was calculated as V = (L × W × W)/2. VEN = venetoclax. ****p < 0.0001. (B) Body weights during the treatment period.

**
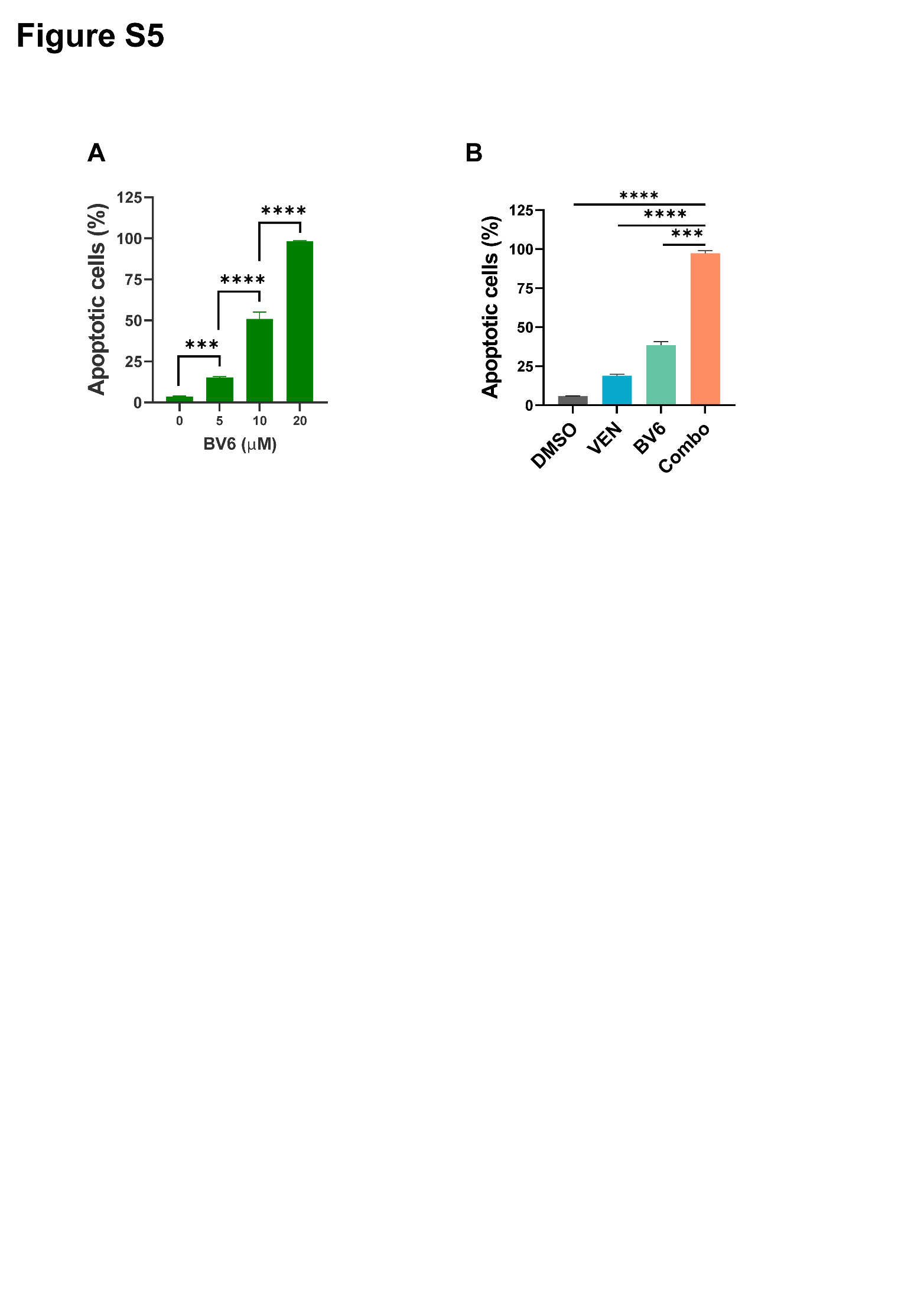
**

**Figure S5.** The anti-lymphoma efficacy of BV6 against the aggressive PT14 cells. (A) PT14 cells were treated with BV6 at indicated concentrations for 24 h, then annexin V/PI apoptosis assay was performed to determine the percentage of apoptotic cells. (B) Apoptosis detection was conducted after 24-h treatment of PT14 cells with BV6 (10 µM), venetoclax (0.5 µM) or their combination.


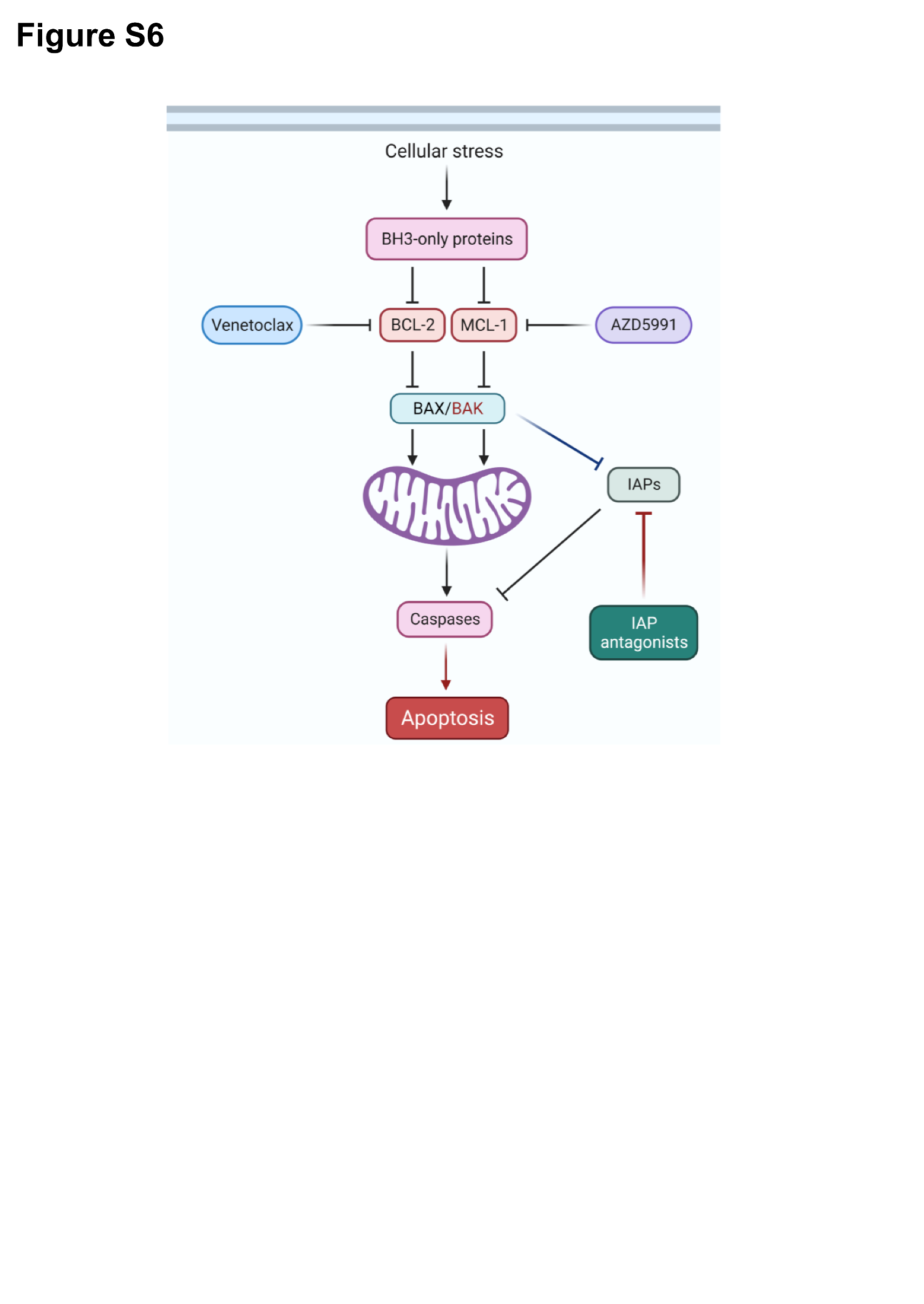


**Figure S6.** Schematic diagram of the mitochondrial apoptosis pathway and mechanisms associated with AZD5991 (Created with BioRender.com).
