# Supplementary material for: Potentiation of apoptosis in drug-resistant mantle cell lymphoma cells by MCL-1 inhibitor involves downregulation of inhibitor of apoptosis proteins
Source: Cell Death Dis. 2023 Nov 2;14(11):714. doi: 10.1038/s41419-023-06233-w (PMC10622549; doi:10.1038/s41419-023-06233-w)

Figure 1F

Western blot analyses after 24-hour treatment of AZD5991 at indicated concentrations

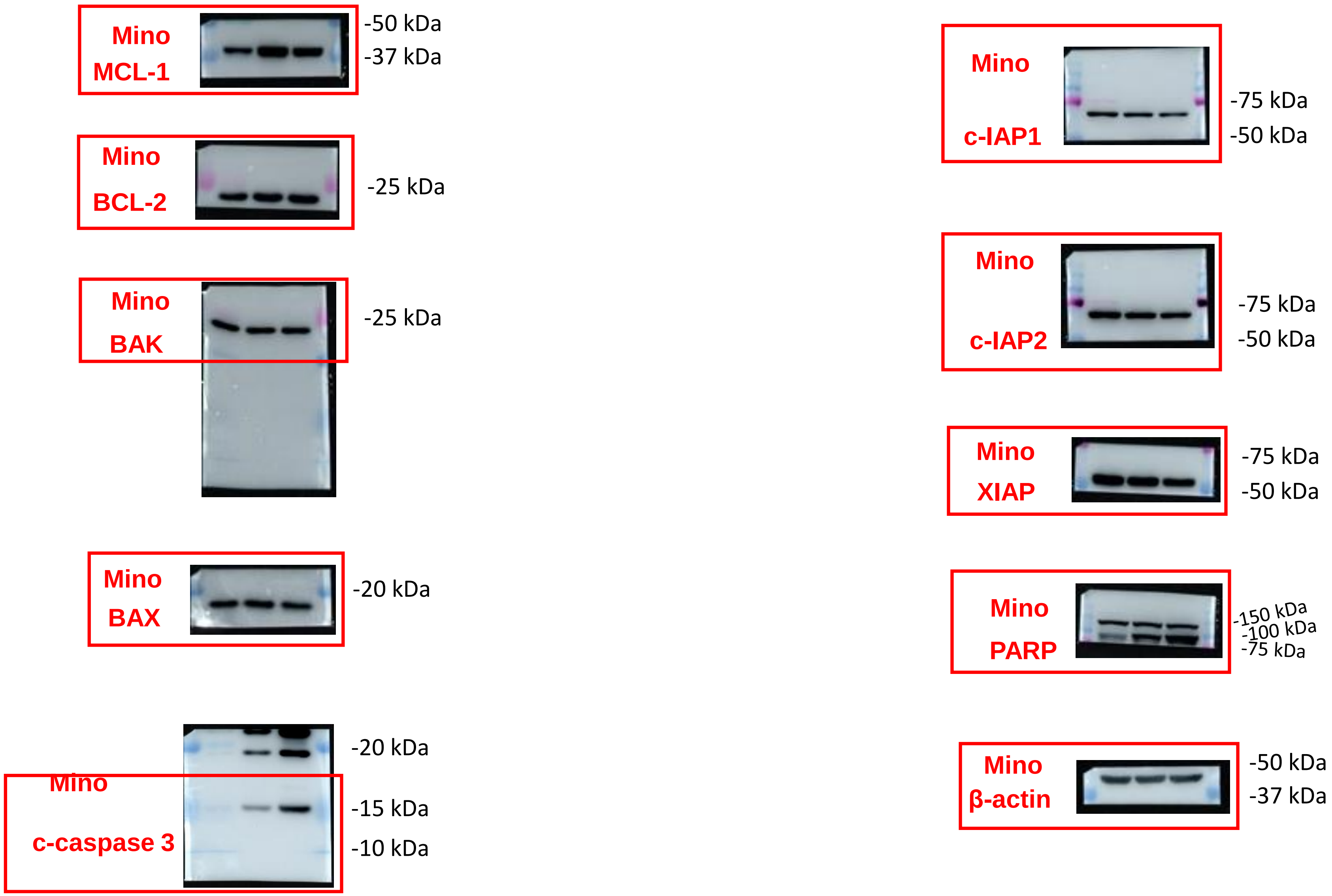

Figure 1F

Western blot analyses after 24-hour treatment of AZD5991 at indicated concentrations

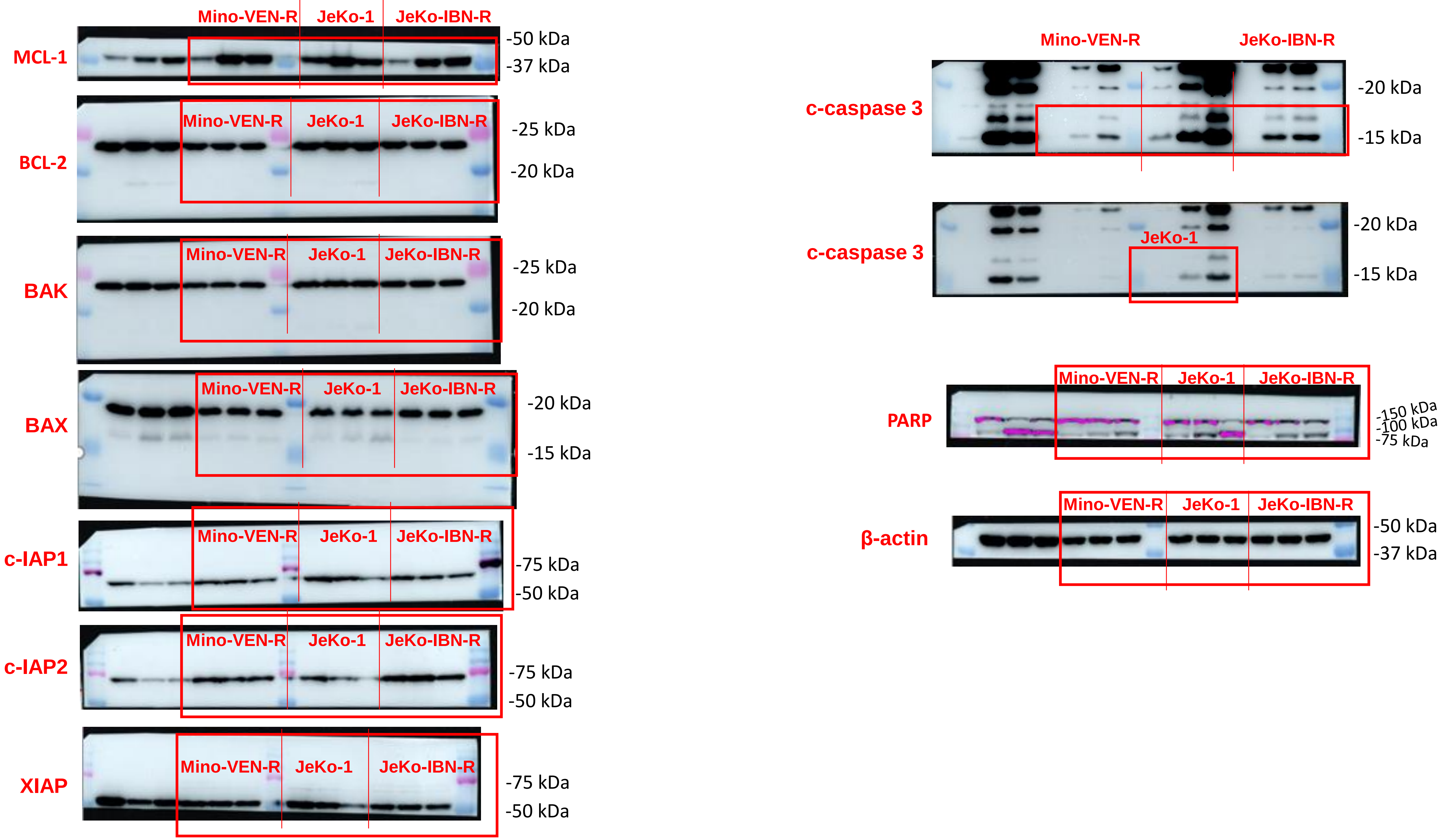

Figure 1G

IAPs expression in Mino cells treated with two additional MCL-1 inhibitors, S63845 and MIK665 for 16 hours

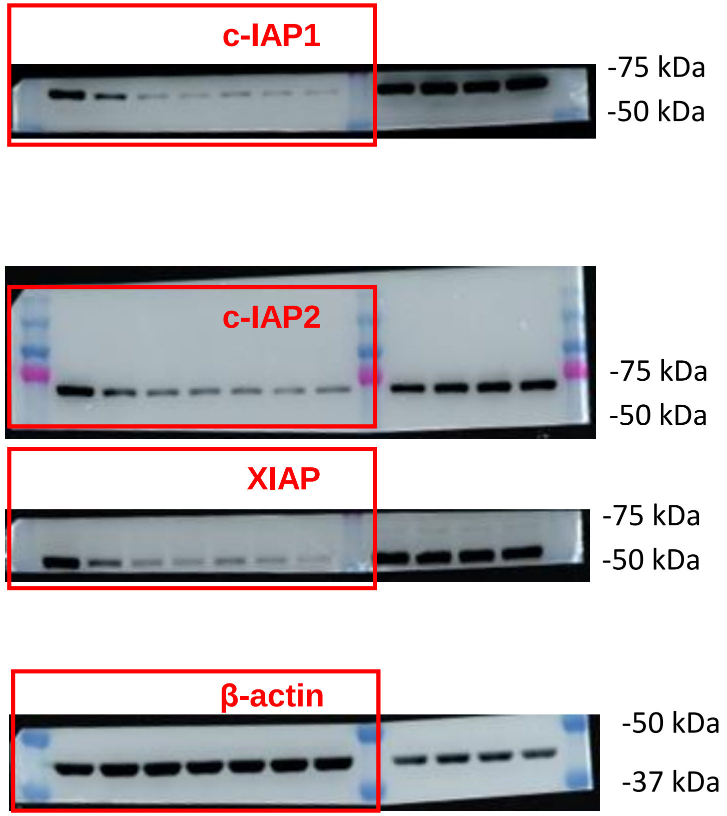

Figure 1H

Expression of IAPs in Mino cells following electroporation with three MCL-1 siRNAs

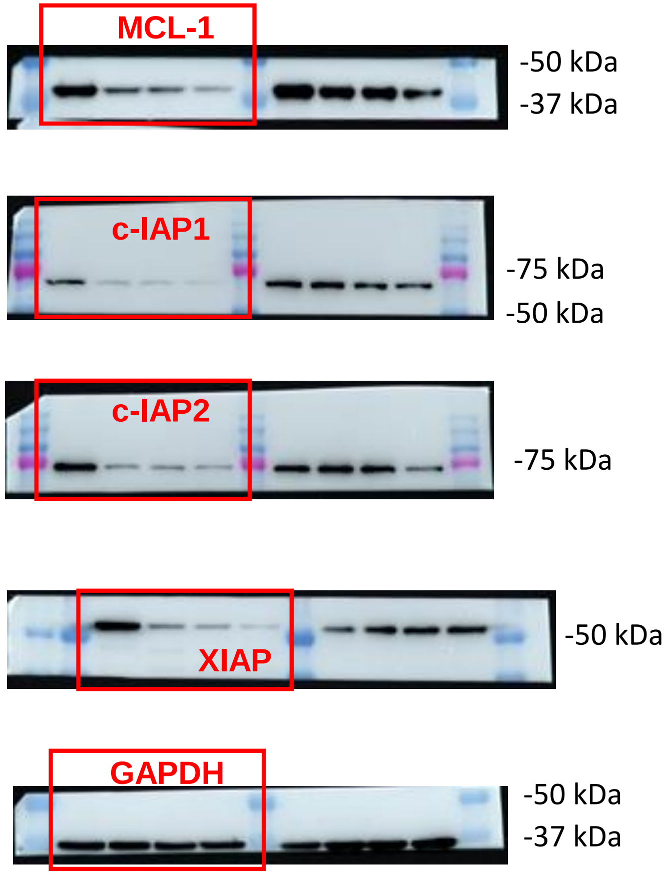

Figure 2A

Whole cell extract (bottom) and co-IP (top) of MCL-1 from lysate of after a 15-minute treatment with AZD5991 in JeKo-1, JeKo-R, or JeKo BTK KD_2 cells

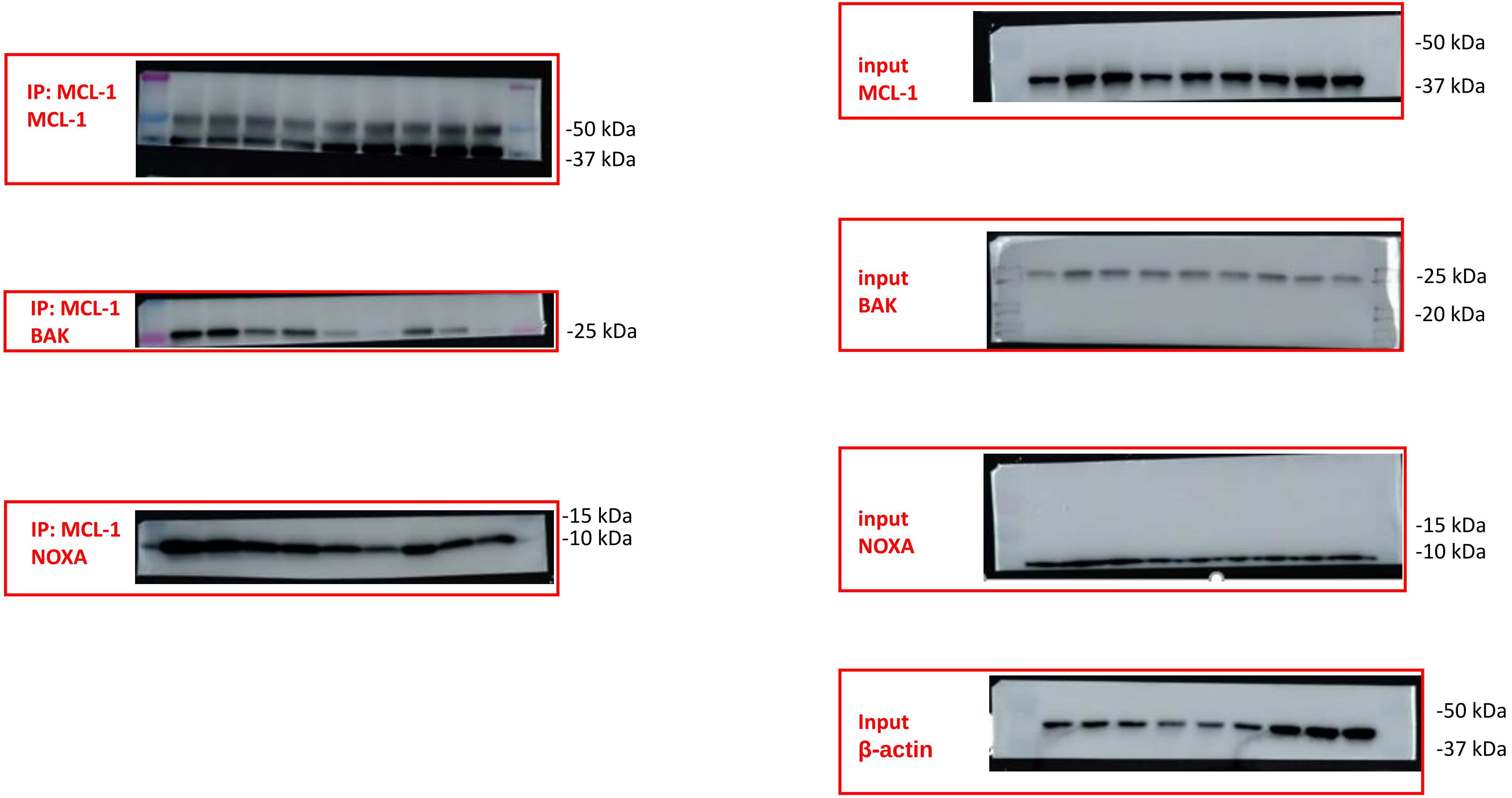

Figure 2B

Time-dependent co-IP assay of MCL-1 with AZD5991 treatment in JeKo BTK KD_2 cells

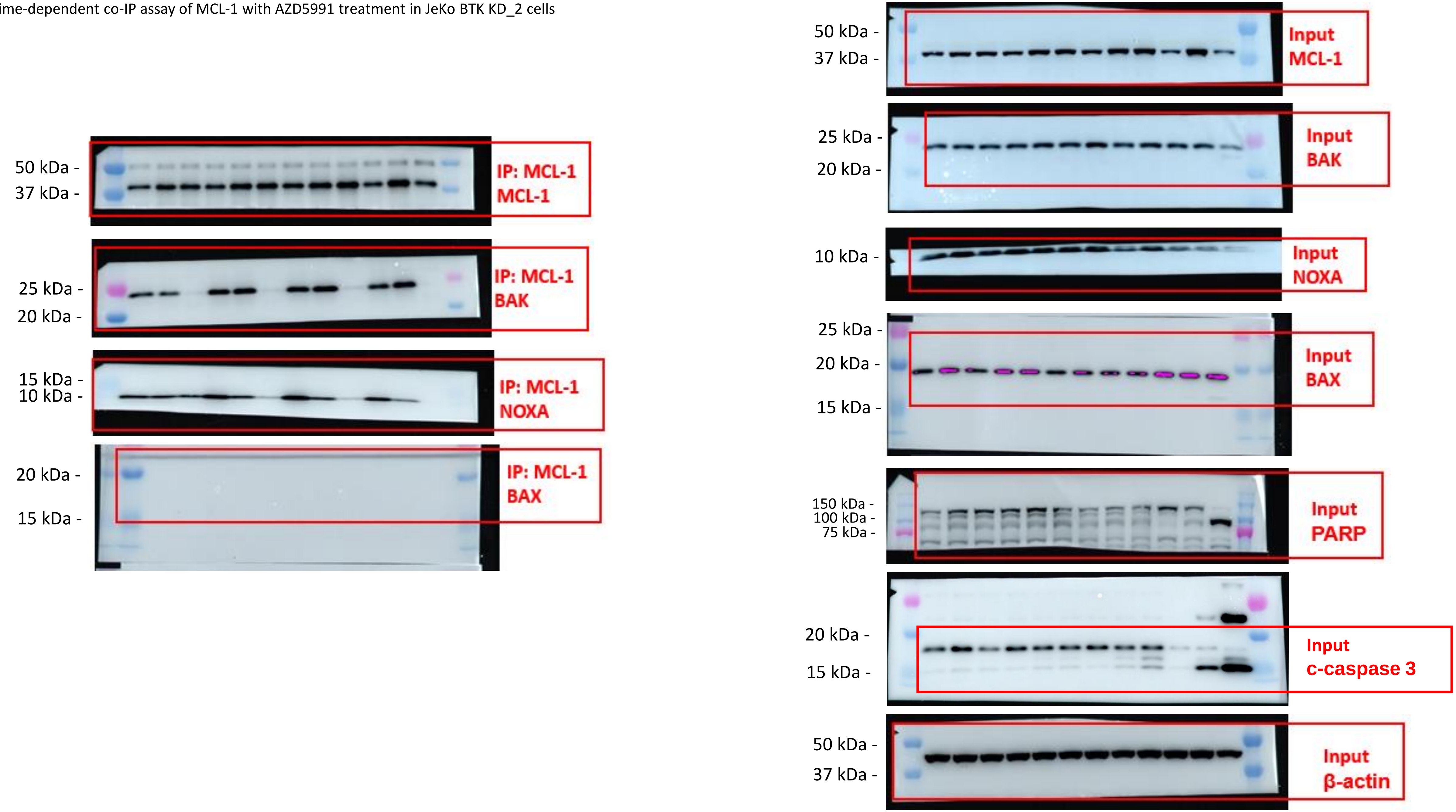

Figure 2C

JeKo-1_ JeKo-IBN-R siRNA_BAK

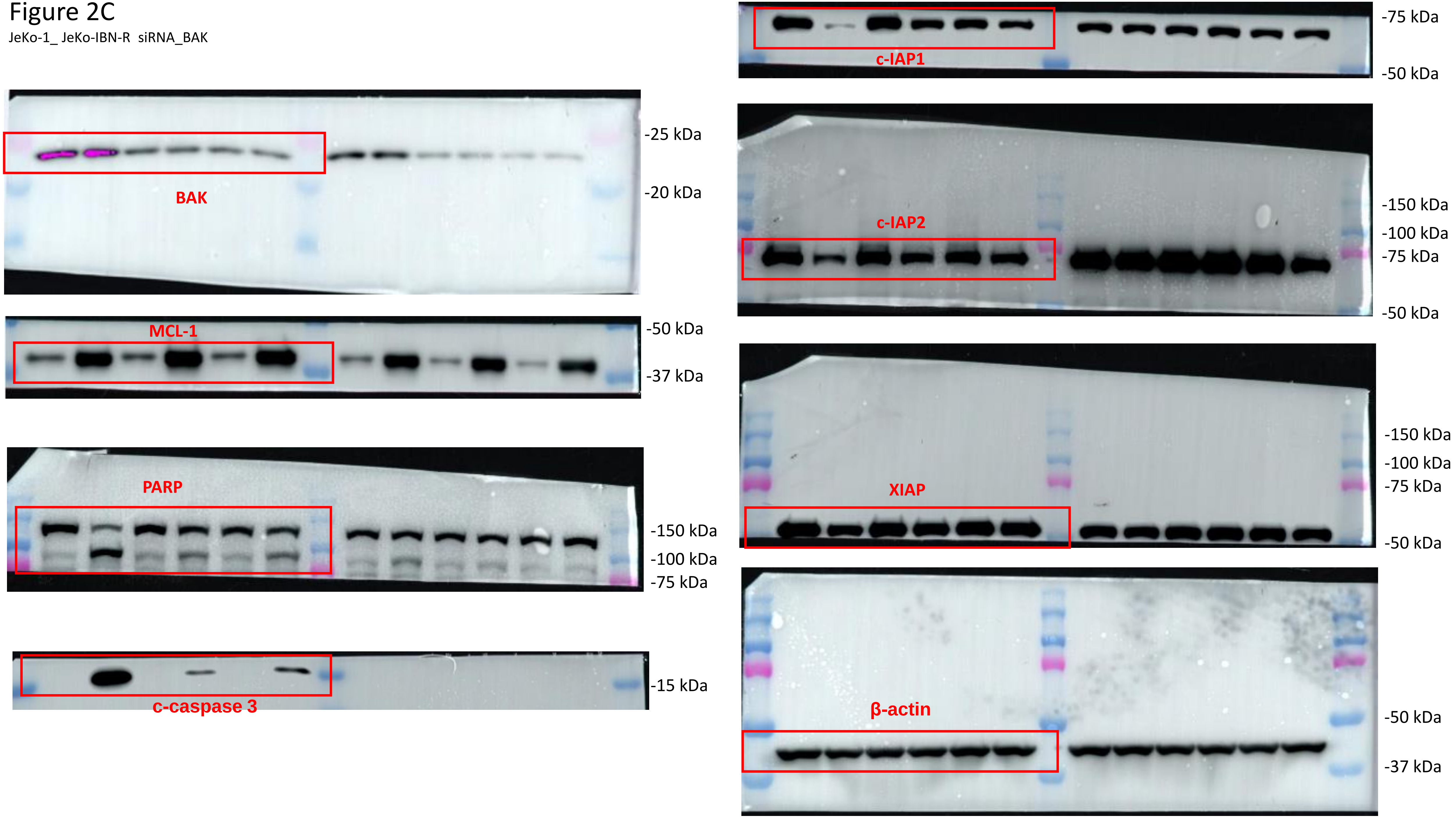

Figure 3C

Western blot analyses in JeKo-1, JeKo-IBN-R, Mino and Mino-VEN-R cells treated with AZD5991, venetoclax, or the combination for 16 hours.

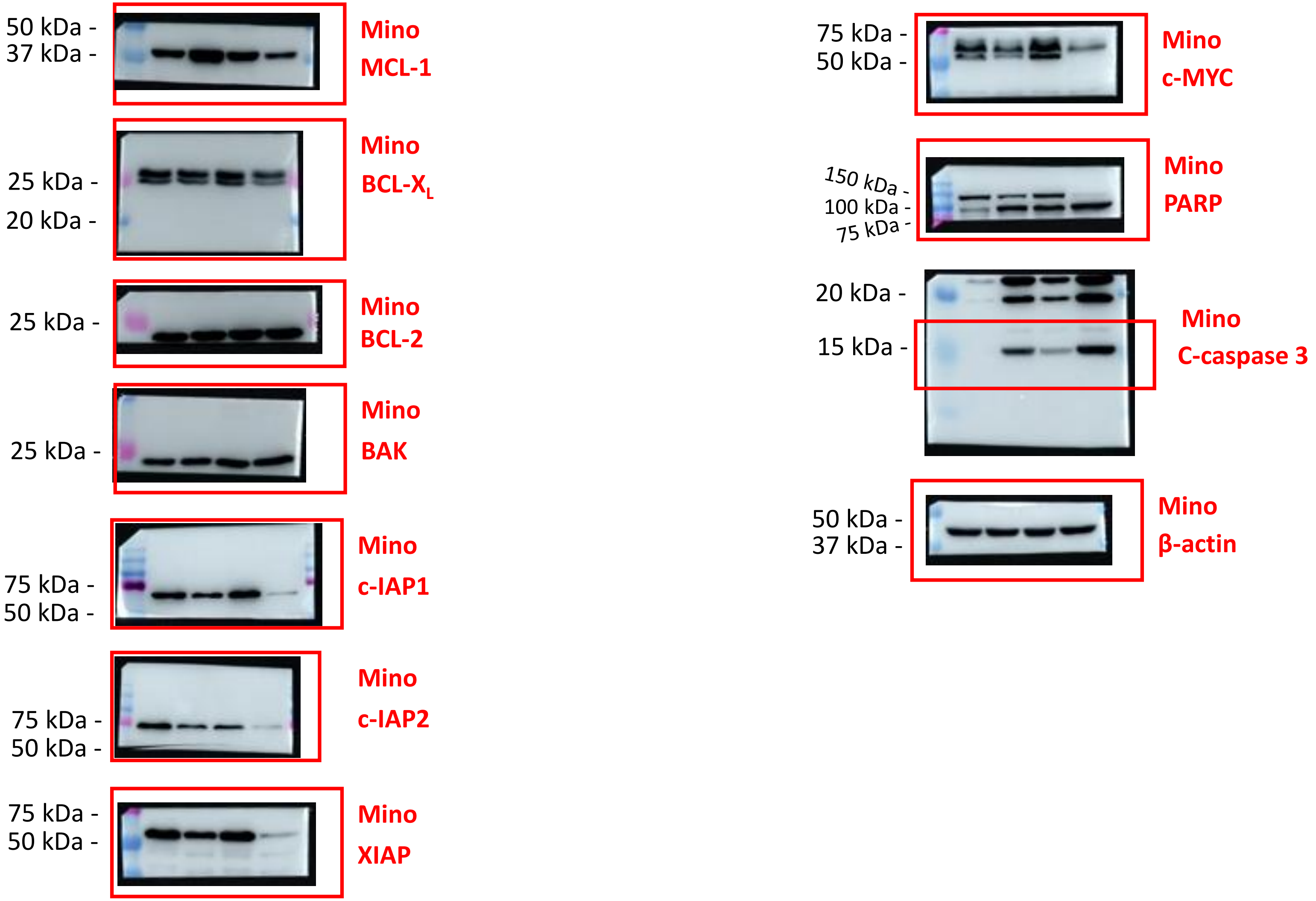

Figure 3C

Western blot analyses in JeKo-1, JeKo-IBN-R, Mino and Mino-VEN-R cells treated with AZD5991, venetoclax, or the combination for 16 hours.

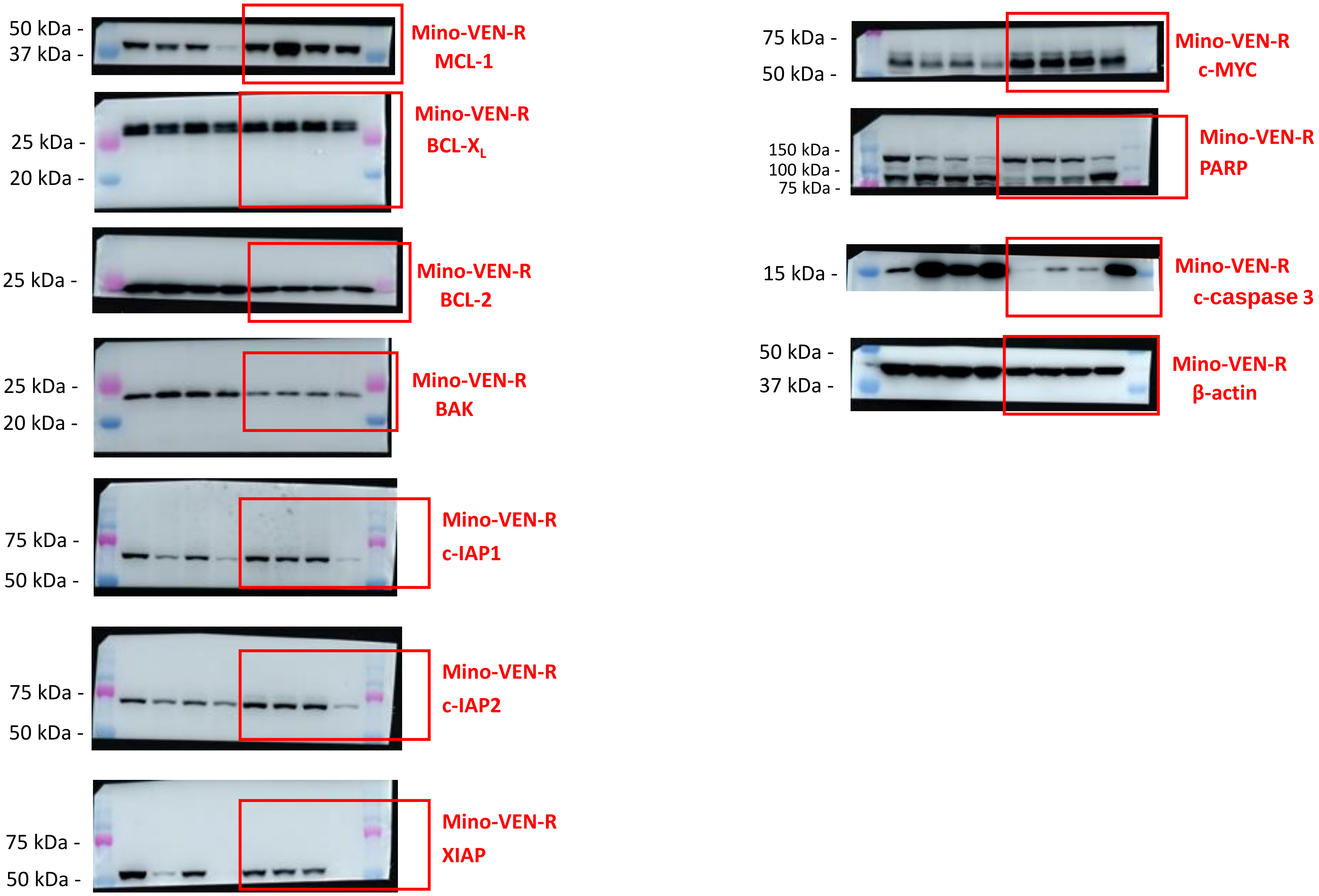

Figure 3C

Western blot analyses in JeKo-1, JeKo-IBN-R, Mino and Mino-VEN-R cells treated with AZD5991, venetoclax, or the combination for 16 hours.

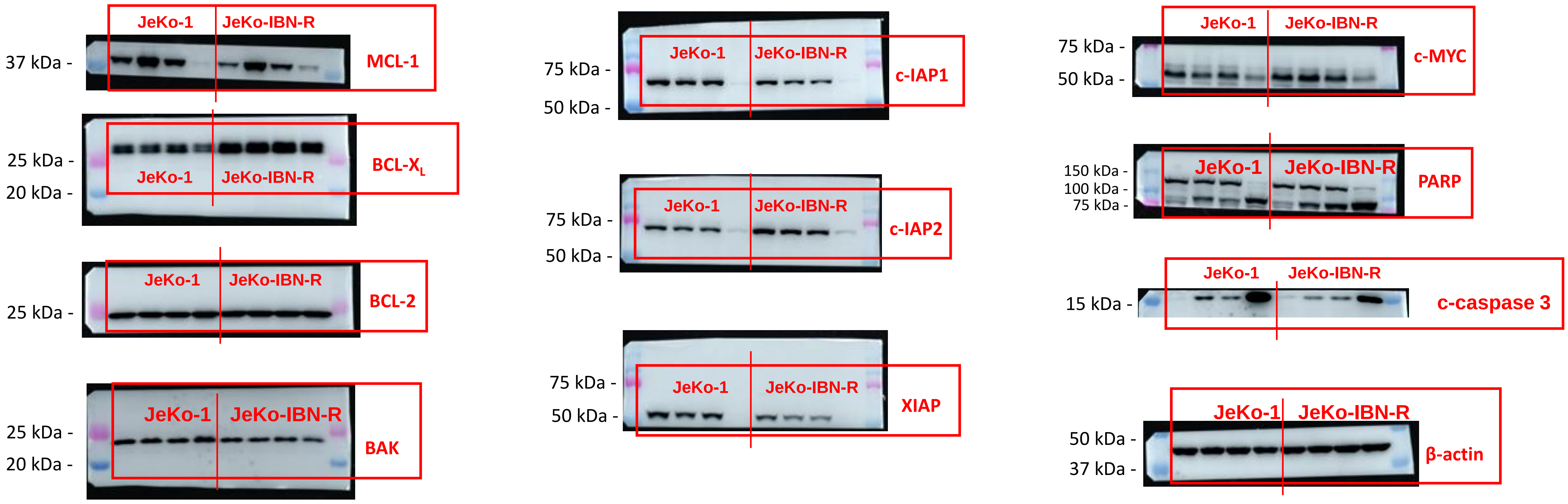

Figure 4D

Expression of c-Myc and pro-apoptotic markers were detected by western blot after 4-hour and 24-hour treatment of AZD5991 (0.2 µM) and venetoclax (0.2 µm) against PT14 cells.

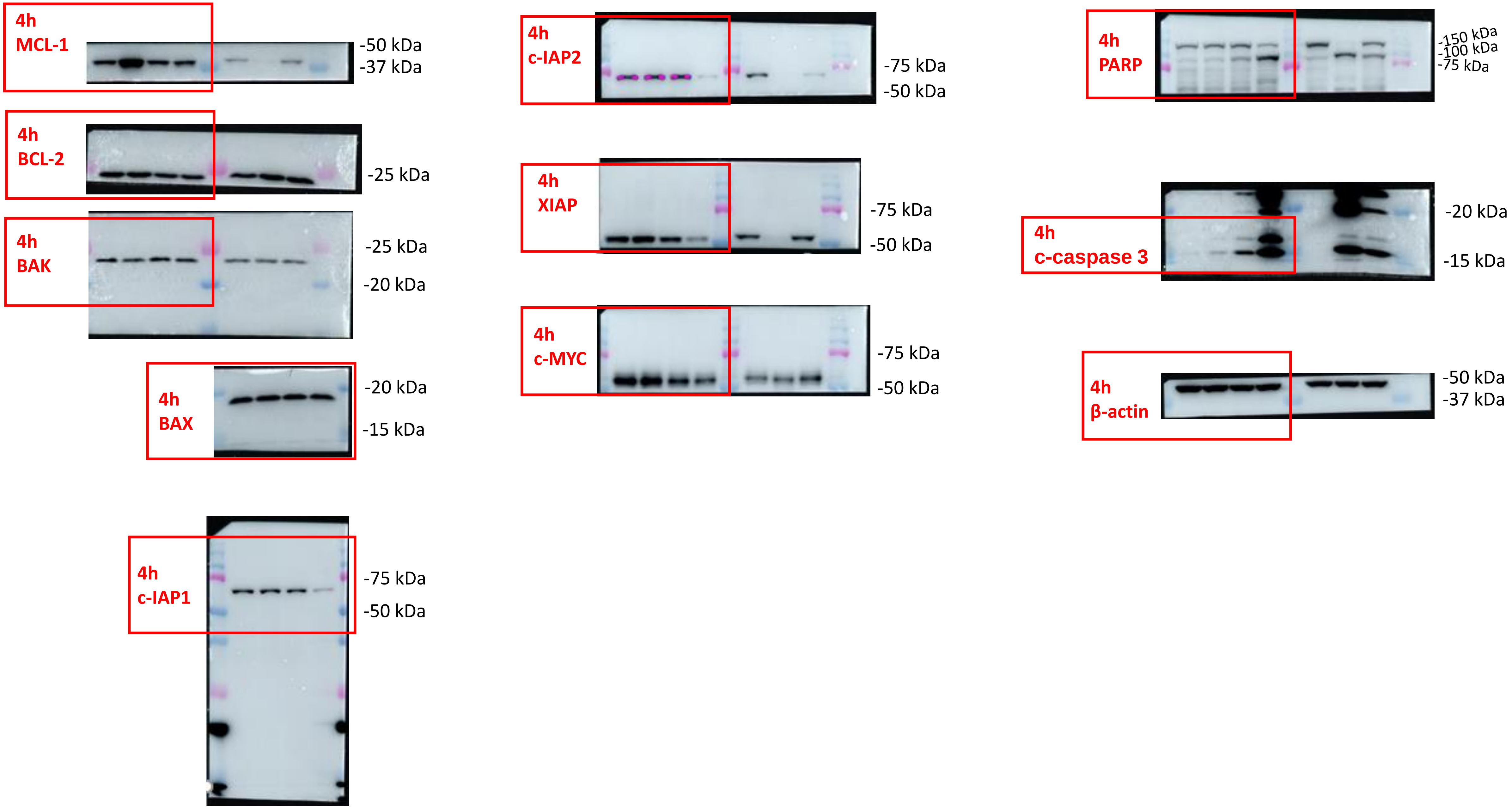

Figure 4D

Expression of c-Myc and pro-apoptotic markers were detected by western blot after 4-hour and 24-hour treatment of AZD5991 (0.2 µM) and venetoclax (0.2 µm) against PT14 cells.

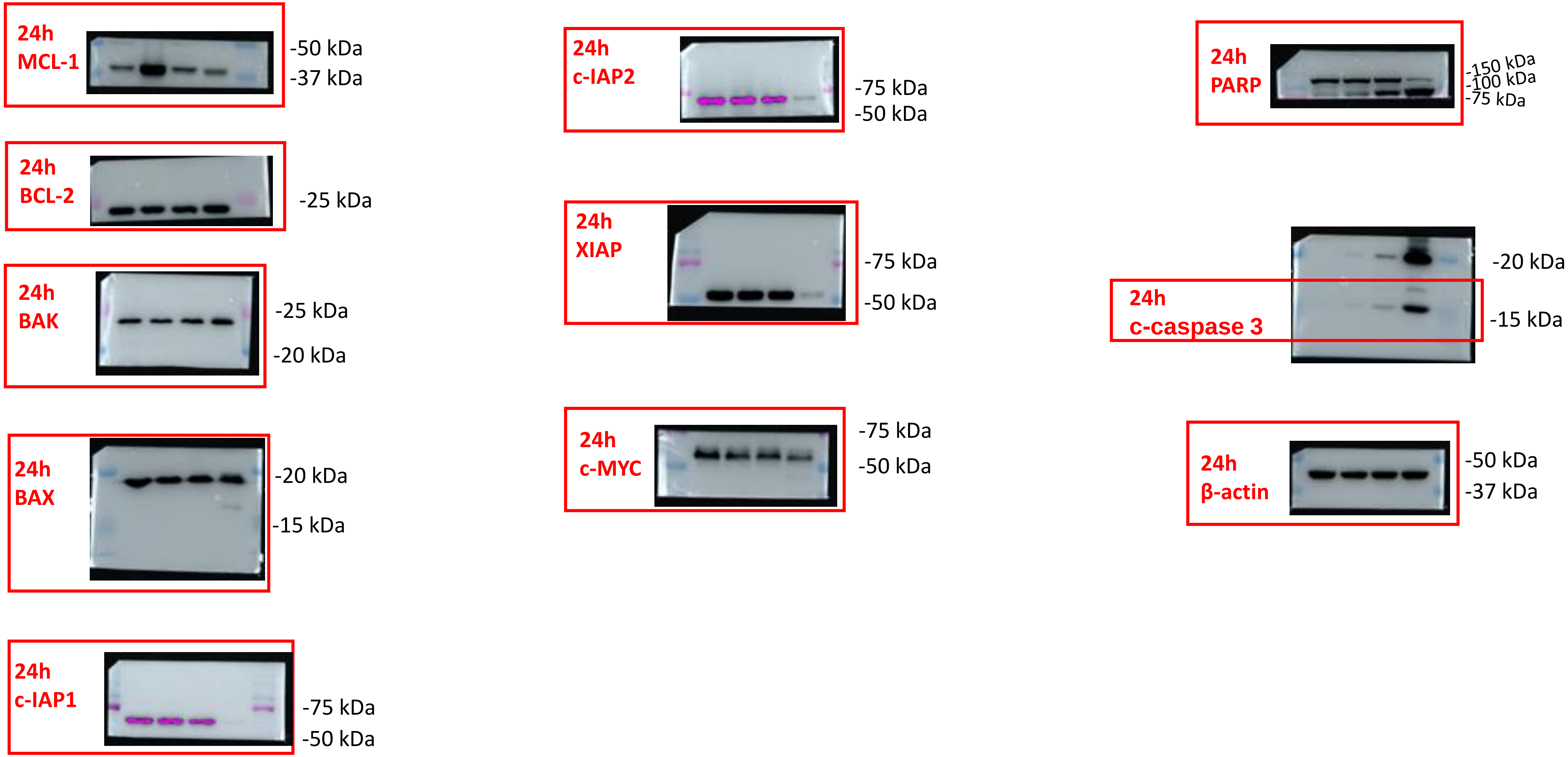

Figure 7A

Western blot analyses of IAP family proteins across MCL cell lines.

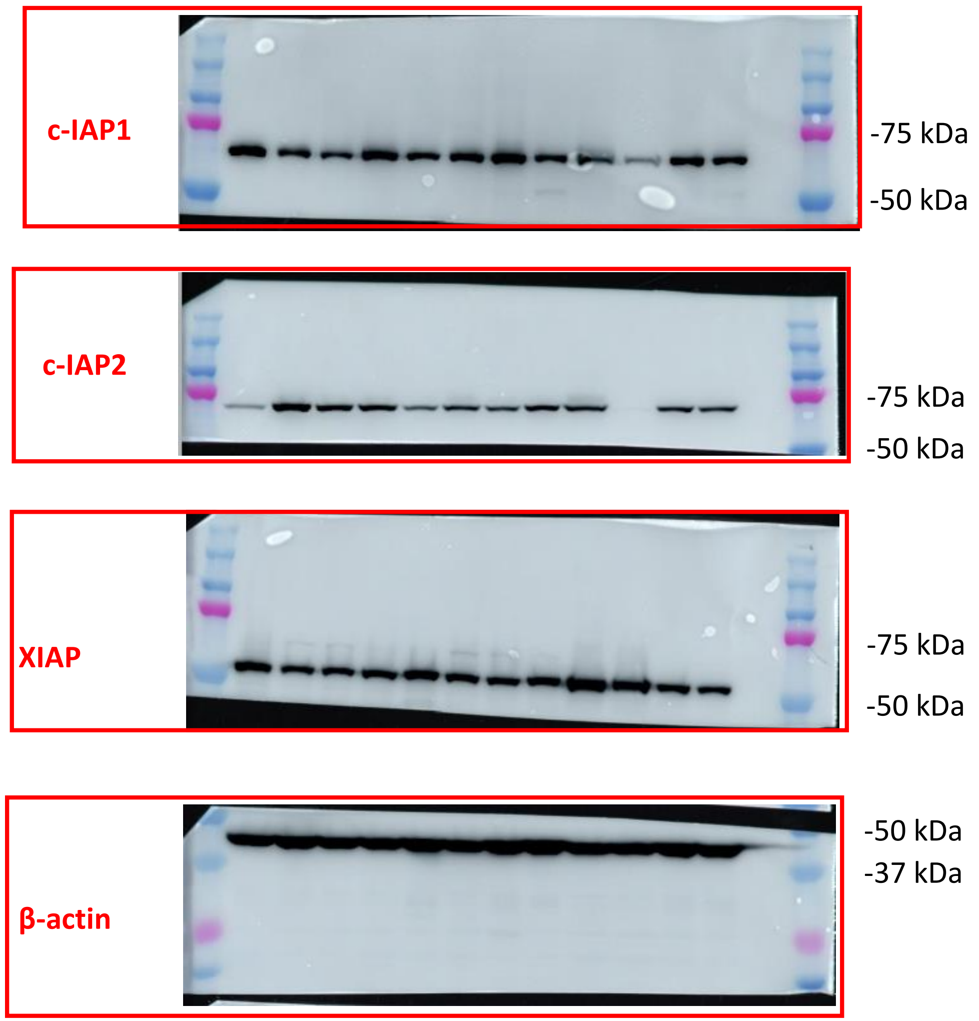

Figure 7C

MCL cell lines were treated with BV6 at indicated concentrations for 4 hours, followed by detection of the IAP expression

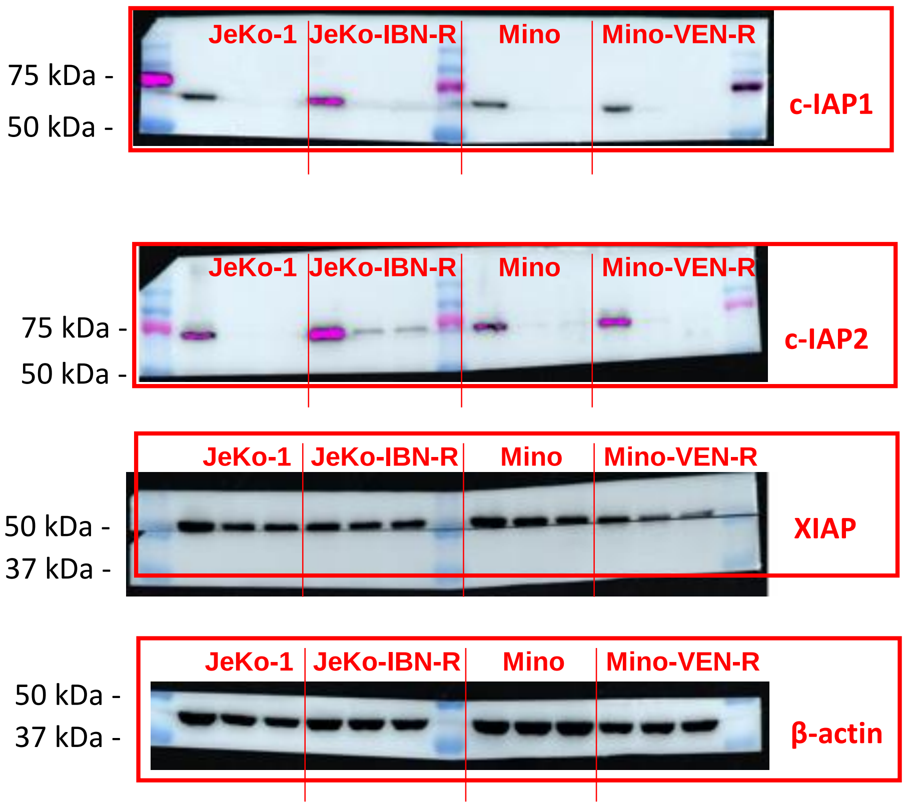

Figure 7E

Expression of IAPs and apoptosis-related proteins were measured after 16-hour treatment of the BV6 (10 μM) and venetoclax

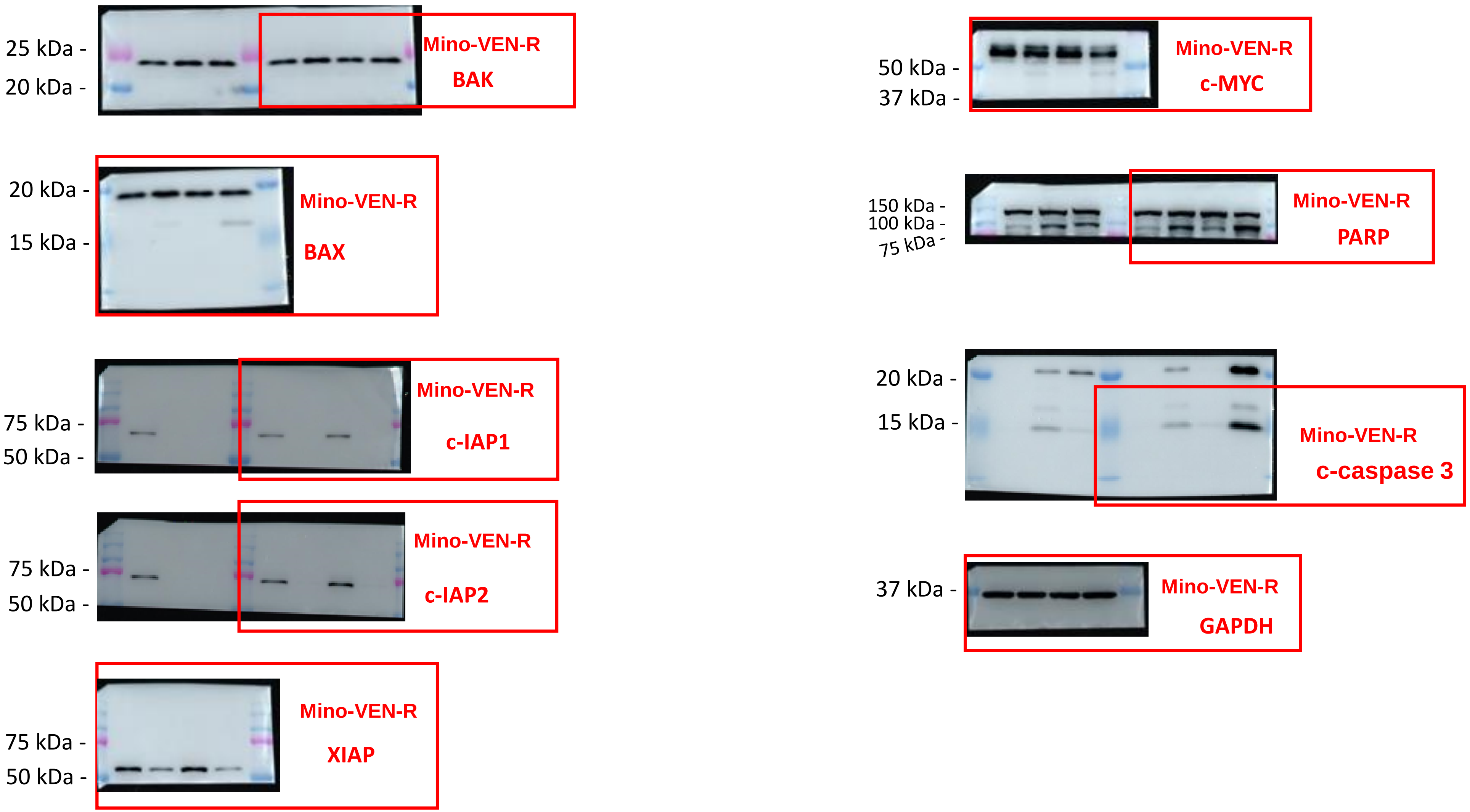

Figure 7E

Expression of IAPs and apoptosis-related proteins were measured after 16-hour treatment of the BV6 (10 μM) and venetoclax

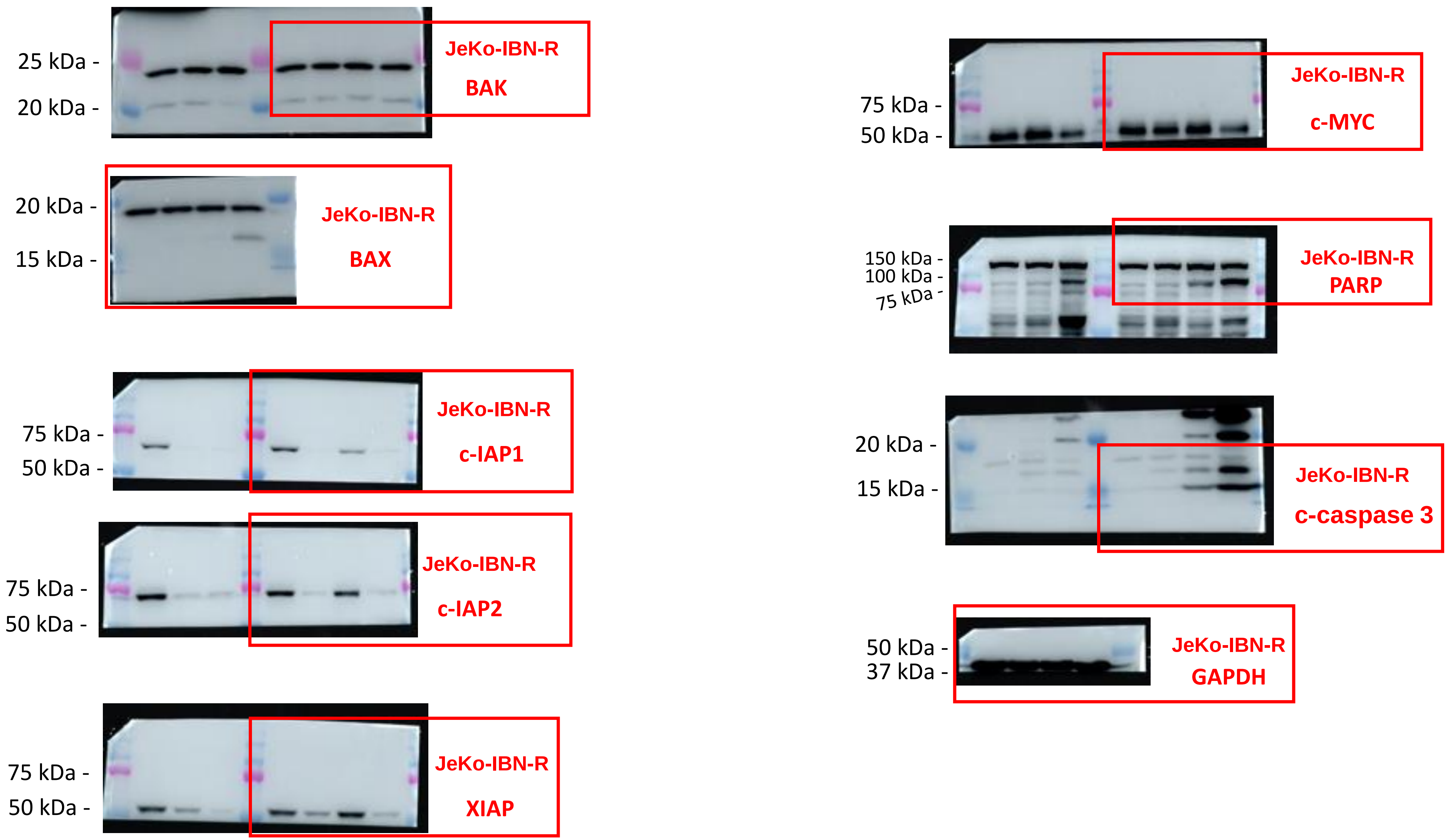

Figure S1A

Expression levels of IAPs in JeKo-1 cells following treatment with two additional MCL-1 inhibitors

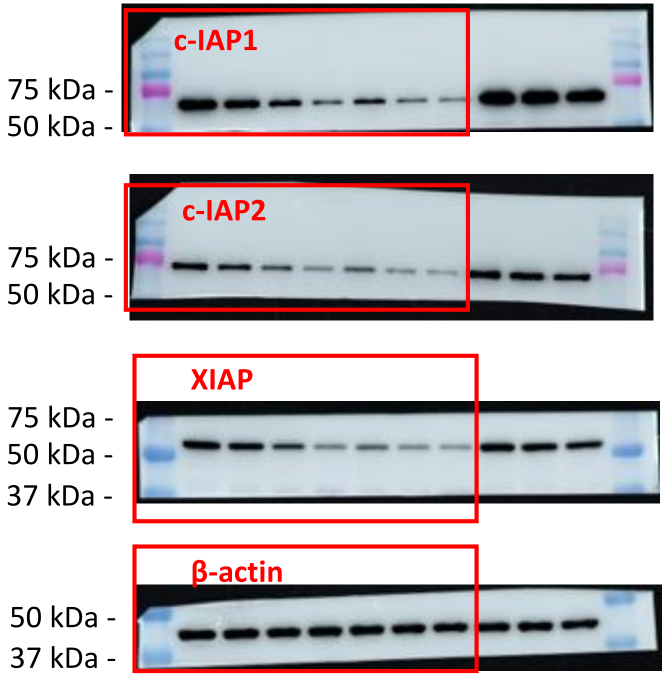

Figure S1B

Expression levels of IAPs in JeKo-1 cells following treatment with siRNA by electroporation

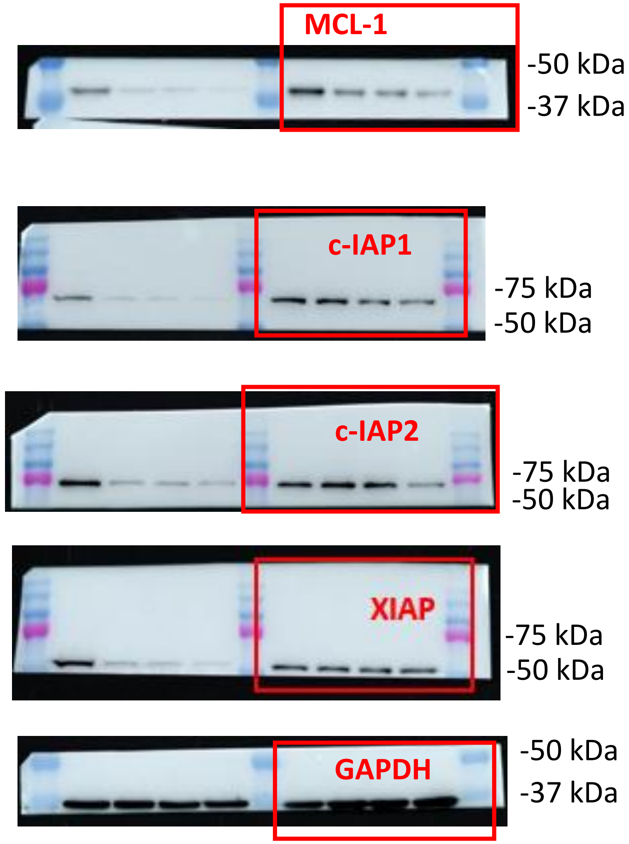

Figure S2A and S2B

Co-IP analysis of MCL-1 in JeKo-1 cells after 2- or 4-h treatment with AZD5991 at indicated concentrations

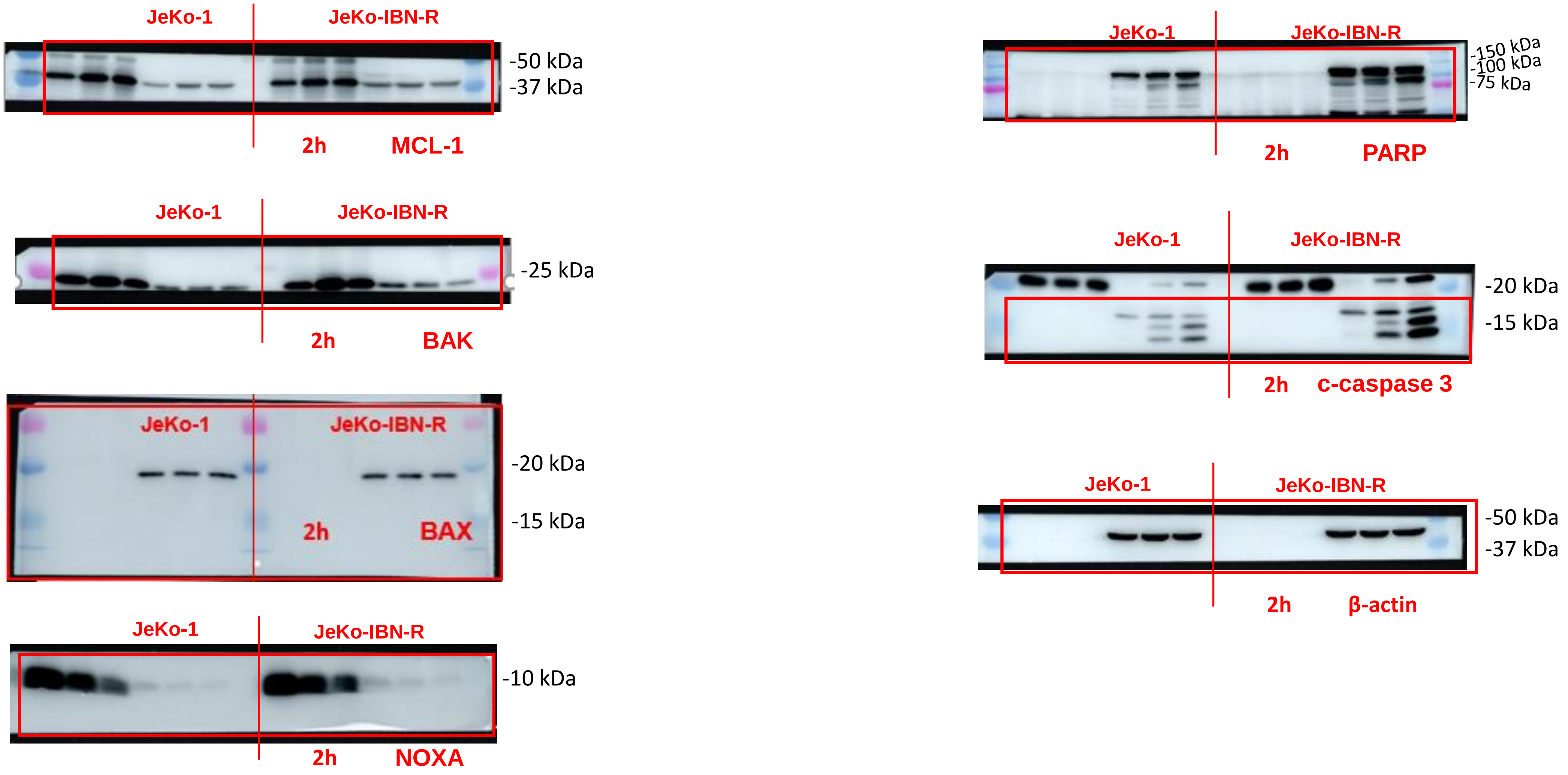

Figure S2A and S2B

Co-IP analysis of MCL-1 in JeKo-1 cells after 2- or 4-h treatment with AZD5991 at indicated concentrations

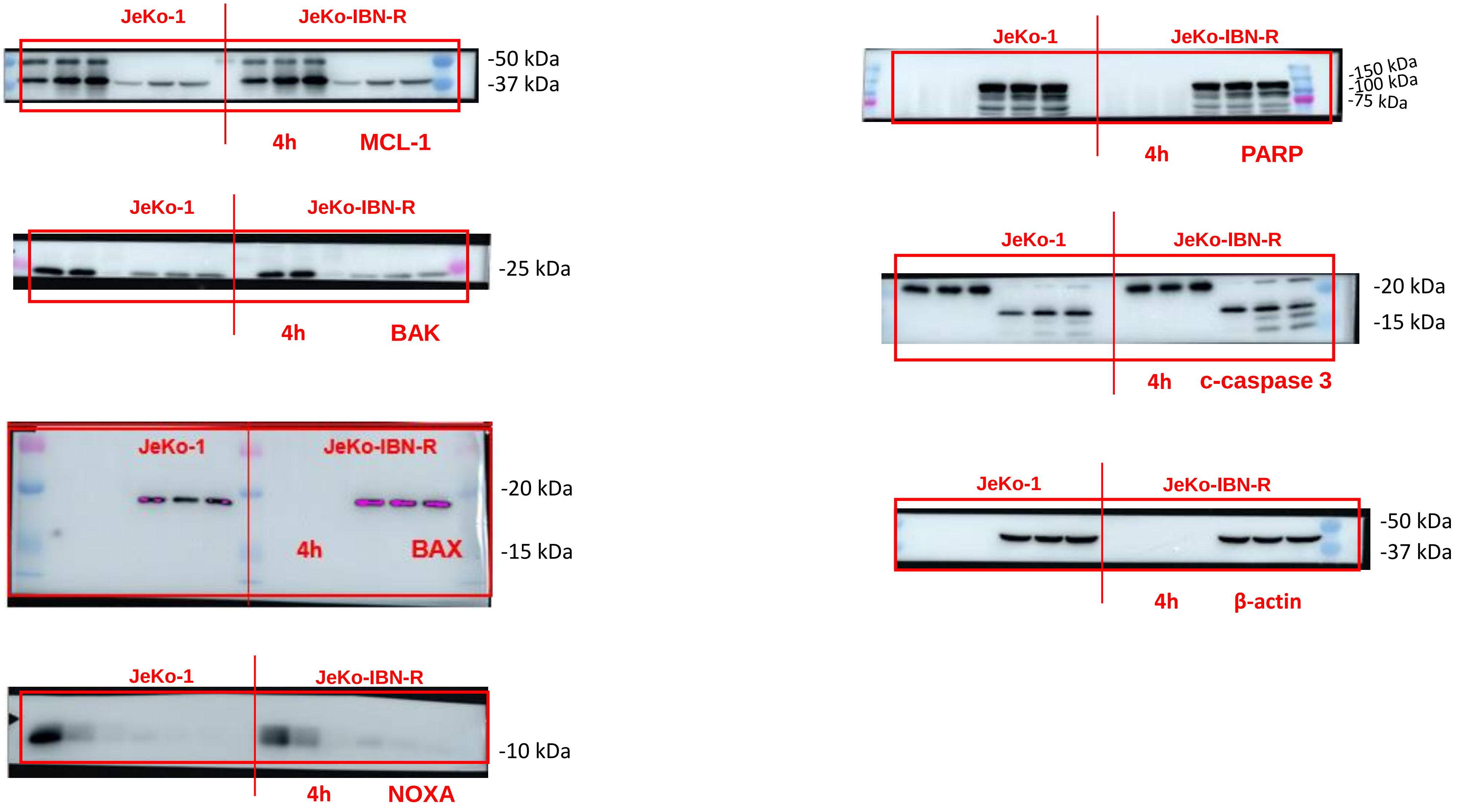

Figure S2C

Co-IP with MCL-1 in JeKo-1 and JeKo BTK KD_2 cells

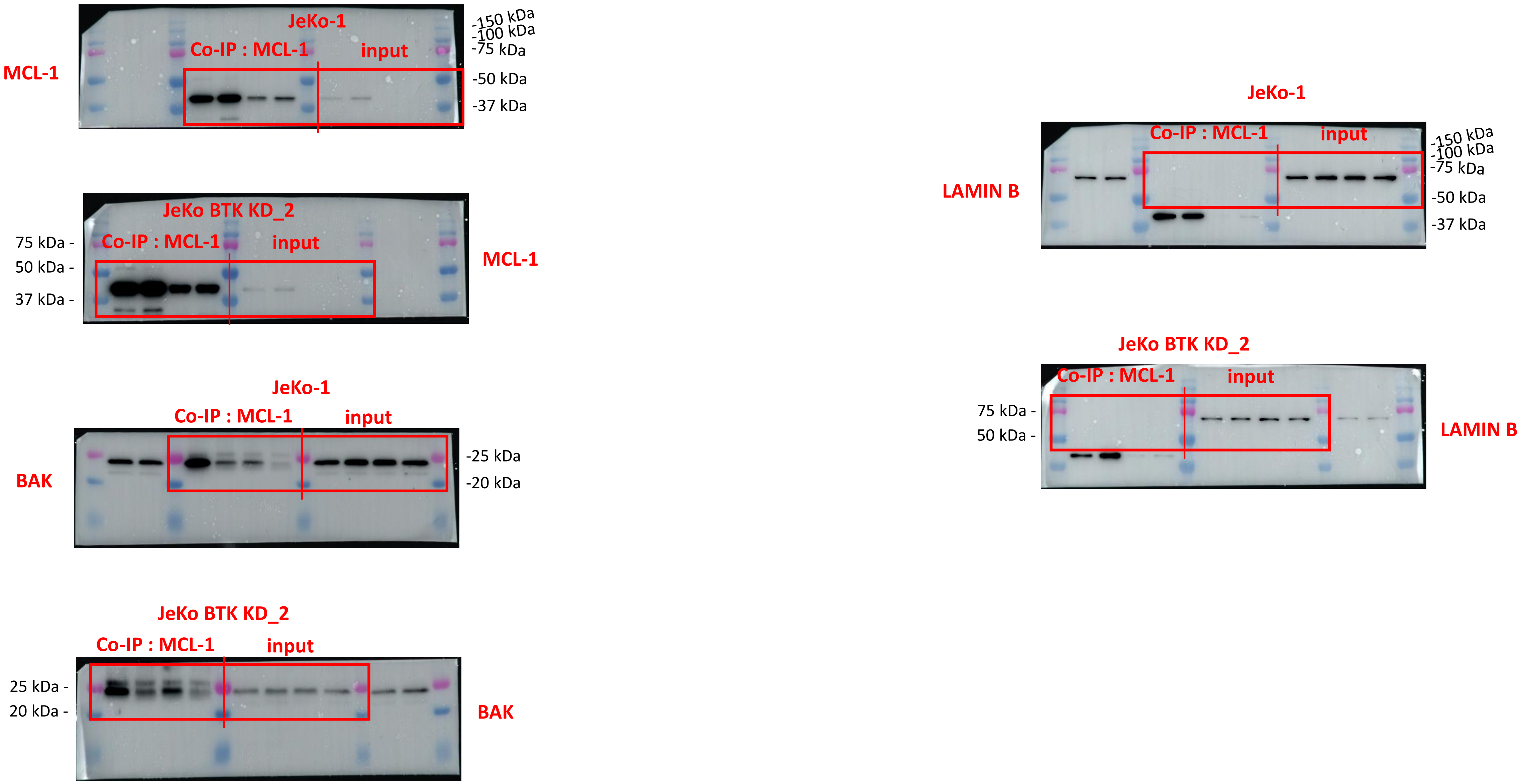

Figure S2D

Co-IP analysis of BAX in JeKo BTK KD_2 cells

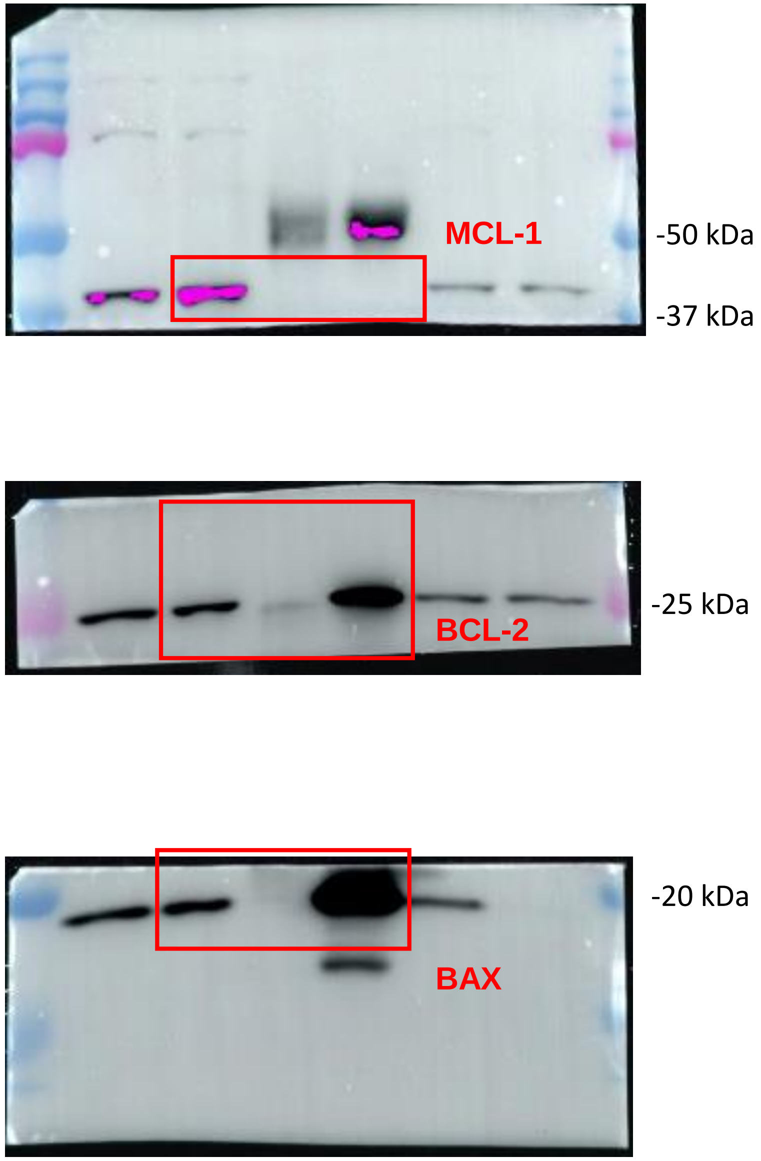

Figure S3A

siRNA_Bak in Mino cells, OCR_Sea horse

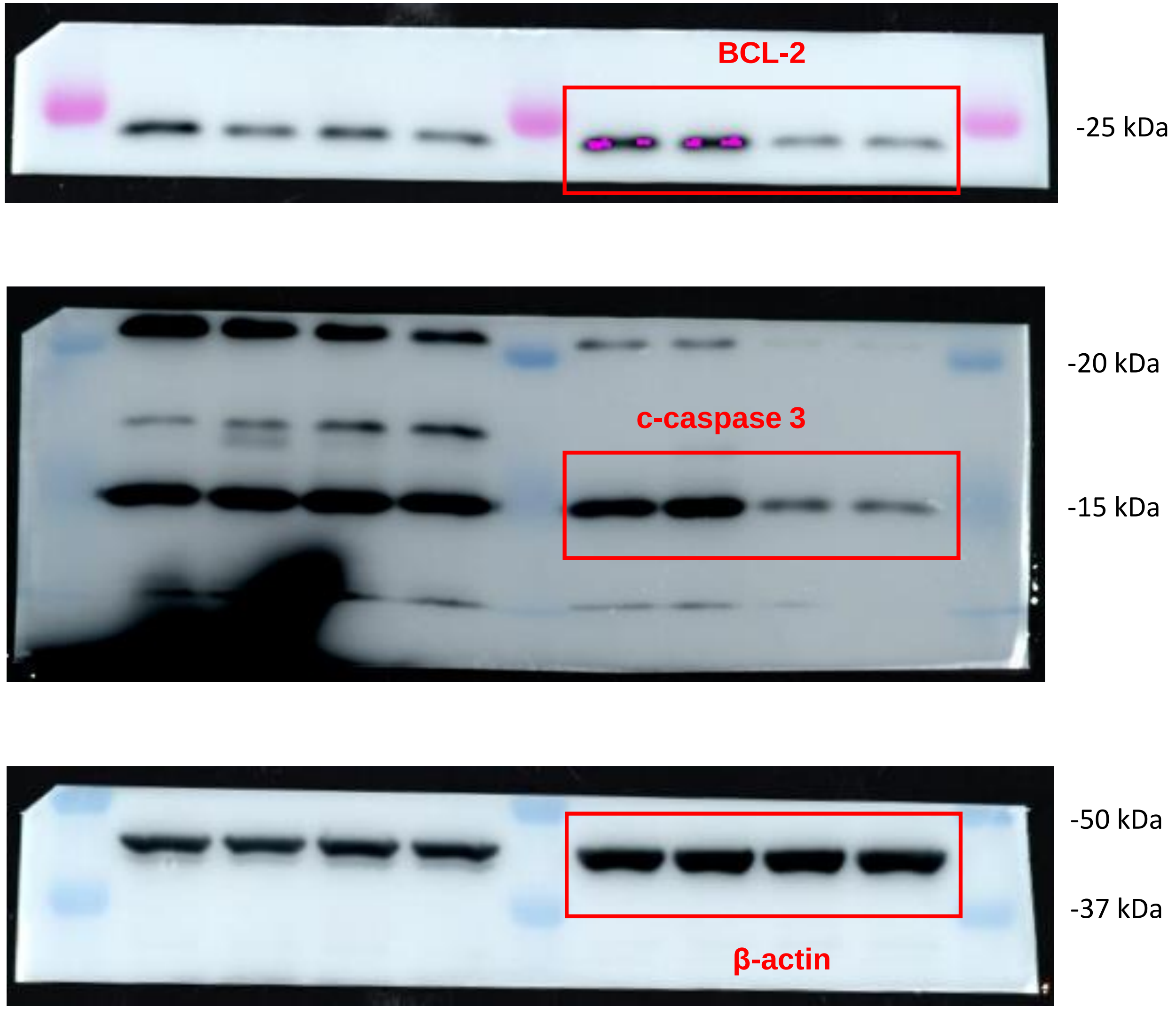

Supplement: Supplementary file 2 — Original Data File [file 41419_2023_6233_MOESM2_ESM.pdf]
